# Analectic

**Published:** 1837

**Authors:** 


### MISCELLANEOUS INTELLIGENCE.

### ANALECTIC, ANALYTICAL, AND ORIGINAL.

### 1.—ANALECTIC.

### AD SYLVAS NUNCIUS.

1. *Physiology.—Experiments on the mechanism of the motion or beating arteries.* By M. FLOURENS.*—Connected with the mechanism of the motion of arteries, two questions present themselves for consideration, the cause which produces it, and the mode in which it takes place. M. Flourens considers these separately.

1. *The cause.*—Galen's opinion, that it depended upon a pulsific power, was founded upon the following experiment. Into the interior of an artery opened longitudinally, a tube was introduced, and a ligature placed so as to keep the artery in close contact with it. According to his statement, though the blood flowed into the artery beyond the tube, pulsation ceased. The experiment has been repeated by Vieussens, Magendie, and other physiologists, with an opposite result, and the following observations of M. Flourens corroborate their views. On introducing a goose quill into the aorta of a sheep, opened longitudinally, and fixing it in its situation by ligature, and even when a portion of the vessel was excised and the communication maintained by the quill, the blood was transmitted, and all the arteries connected with it continued to beat. M. Flourens adopts Harvey's opinion, that the cause of the motion of arteries is the impulse communicated by the flood forced into them by the contractions of the heart. He discusses and repels the objections offered to Harvey's doctrine by Lamure, and shows the fallacy of the conclusion drawn from the following experiment. Lamure included between two ligatures a portion of an artery full of blood, and because this portion continued to move, he concluded that the motions of arteries were not produced by the impulse of the blood. The fallacy consists in his overlooking the fact, that the mo-

* From Comptes rendus Hebdomadaires des Seances de l'Academie des Sciences, 23 Janvier 1837.

tion observed was communicated fiom the artery above the ligature, and was not proper to the artery.

2. *Mode.*—Galen believed this to be by successive dilatations and contractions. Harvey cut through an artery previously isolated, and, taking the cut end in his fingers, perceived it dilating at each pulsation. Weitbrecht, struck with the difficulty of explaining the whole motion of an artery by its dilatation and contraction, supposed that in addition, displacement or locomotion of the whole vessel took place. Lamure attributed it to elevation of the artery, and imagined that when he put one finger below and another above it, it started during pulsation so as to strike the latter. Arthaud having rendered the arteries of the mesentery straight in several animals, saw or believed that he saw the pulsation, which occurred when the arteries were curved, cease when they were straightened. M. Flourens repeated these experiments. To that of Harvey he objects, that it is very difficult to distinguish the pressure of the artery against the fingers, occasioned by the blood from its natural dilatation. Lamure has not stated correctly the result of his experiment for when repeated by M. Flourens, the artery struck the finger placed above and that placed below it. Arthaud's experiment is unsatisfactory, inasmuch as, in drawing out the arteries to render them straight, they are weakened, and after all their motion does not altogether cease. The experiment of Harvey being then insufficient, that of Lamure inaccurate, and that of Arthaud incomplete; further inquiries appeared to be necessary. To determine the point, M. Flourens determined to investigate experimentally the different elements which concur in producing the motion of arteries, such as dilatation, locomotion, &c. Since the time of Bichat, who adopted the opinion of Weitbrecht, almost all physiologists have explained the pulsation of the arteries by joining dilatation to displacement. Magendie has proved the dilatation of the vessel, and M. Poiseuille has invented an instrument to demonstrate it, though at the same time it shows this dilatation to be by no means considerable. To pursue the enquiry, M. Flourens contrived some instruments to exhibit any change which might take place in the diameter of an artery. These consist of small rings made of watch-spring divided at one point. These are formed in such a way, that when they embrace the artery both ends meet and no more. They had sufficient flexibility to yield on the smallest dilatation of the vessel, and when this ceased, their elasticity caused them to close. The smallest dilatation, then, of an artery will be appreciable, and they have this further advantage, that, from their being capable of being opened, they can be easily applied; and being of all sizes, one can be selected which will exactly embrace the artery, a condition quite indispensable for the accuracy of the experiment. One of these being applied to the abdominal aorta of a rabbit, the cut ends appeared to be alternately separated and approximated. This was repeated several times on different rabbits and always with the same result. On applying one of these rings to the abdominal aorta of a dog, the effect was more distinct, as might have been expected from the greater size of the vessel. From what has now been stated, it is evident that the dilatation does take place during pulsation. To ascertain whether displacement takes place, and whether this is in direct proportion to the curvature of the vessel, M. Flourens began by examining what occurs at the angles or flexuosities of arteries. Here he observed a rise and fall, especially at the arch of the aorta, which was raised from the vertebal column. In the mesenteric arteries it was still more distinct, for these being in a great measure free or at least merely sustained by a fine membrane, the displacement can be the better observed. By increasing the curvatures the displacement was increased, and *vice versa*. The same was observed in the straight arteries, though to a less extent. To ascertain whether the artery was elongated, M. Flourens marked with colour a point of the primitive carotid, and, placing a needle below it, observed it alternately advance and retire. Thus he concludes, that, in the pulsation of an artery, there are three primitive actions, dilatation, displacement, and elongation. It became desirable to ascertain to what physical quality of the organ these actions are due. M. Flourens thinks that it is elasticity. Bichat, Sir E. Home, D. Blainville, Beclard, and Chevreul have made us acquainted with the peculiar tissue to which this is owing, and Magendie has deduced from it the nature of the jet of blood which escapes from an open artery. According to him the jet continues under the influence of the contraction of the arteries, and is jerked by the contraction of the ventricles. Upon this M. Flourens remarks, that all the actions constituting the compound action of the artery, may be accounted for by the impulse of the blood and the elasticity of the vessel. Supposing the artery to be full, as it is in its ordinary state, each new quantity of blood forced into it by the ventricles cannot proceed without distending it in length and breadth, and bringing it nearer to a straight line, throughout its whole length.

The beating or whole motion of an artery depends on its elasticity. —*Eclectic Med. Jour.*

---

2. *The Saliva.—As indicative of the state of the stomach in health and disease.*—At a meeting of the Academy of Sciences on the 20th of March, M. Dumas made the following report on a work of Dr. Donne, the object of which was the study of the saliva in a state of health, and in diseases of the stomach.

"The author has proposed to subject the saliva to a careful examination, in order to determine whether the nature of this secretion, so essential to the digestive process, varies or remains constant. He has satisfied himself that the saliva, which is ordinarily alkaline, becomes acid in certain cases, apparently in connexion with morbid affections of the stomach and alimentary canal. I have myself been furnished, by the author, with the means of verifying his results, and have in fact seen, with him, the saliva more or less acid in several patients in the hospital of La Pitie.

"In noticing this change, the mind naturally recurs to the theory by which it has been so often attempted to refer the secretions to the properties of the voltaic pile. It is certainly a striking fact, that the secretions are never neuter, but always either acid or alkaline; and this fact has already suggested the idea, that the secreting organs are the poles of a pile, separating the secreted fluids from the blood. If it is so, and if we are bound to regard the secretions as products of a decomposition effected by electric agency, it is difficult to explain how the poles of the pile could be reversed as a consequence of diseased action; yet it is not impossible to conceive that it may be so.

"We know in fact, that it is sufficient to change the exciting fluid of the pile in order to reverse its poles. If, therefore, the phenomenon of secretion is of an electric character, we ought, by changing the condition of the organ which performs the function of the pile, to change the nature of the fluids secreted. This change in the secretions would not, in this theory, imply that the secreting organ was morbidly affected; the disease would be elsewhere; and by discovering the organ which formed its seat, we should discover the seat of the electric action.

"In viewing the diseases in which the saliva becomes acid, one is struck with the dryness of the mouth; and the question arises, whether there can be in these cases a simple suppression of the salivary secretion, and whether the acid liquor which bedews the tongue, can be the gastric juice coming from the stomach. This query was submitted to the author. But as he has seen the pancreatic fluid itself become acid, as he has observed the acid state of the mouth reappear, immediately after the removal of its fluid contents by washing, and especially as he has remarked the saliva to be acid within the salivary glands, it remains indisputable that the saliva may alter in its chemical character, and become acid in certain morbid affections.

"The acid state of the saliva must exert an injurious influence on the teeth, and too much care cannot be taken to obviate this in the numerous cases in which this change occûrs for a transient period.

"The work of M. Donne is conceived in a just spirit of observation, and the facts it contains are exact. It would be very interesting to subject the principal secretions to a similar examination, not only with the view of ascertaing the modifications of which the several secretions are susceptible, but also for the purpose of determining precisely the alterations of the organs, and especially the condition of the great sympathetic nerve. These are inquiries to which no one is better qualified to do justice than M. Donne."—*Ibid.*

---

3. *Experimental researches into the physiology of the human voice.* By John Bishop, Esq.*—"The following are the conclusions deduced by the author from the inquiries which form the subject of the present paper.

"1. The vibrations of the glottis are the fundamental cause of all the tones of the human voice.

"2. The vibrating length of the glottis depends conjointly on the tension and resistance of the vocal ligaments, and on the pressure of the column of air in the trachea.

"3. The grave tones vary directly, and the acute tones inversely, as the vibrating length and tension of the vocal ligaments.

---

* Communicated by P. M. Roget, M. D. Sec. R. S. [Read before the Royal Society.]

"4. The vocal tube is adjusted to vibrate with the glottis by the combined influence of its variations of length and of tension.

"5. The elevation of the larynx shortens the vocal tube; and its depression produces the contrary effect. The diameter and extension of the tube vary reciprocally with the length.

"6. The falsetto tones are produced by a nodal division of the column of air, together with the vocal tube, into vibrating lengths.

"7. The pitch of the vocal organs, when in a state of rest, is, in general, the octave of the fundamental note.

"The paper was illustrated by several drawings."—*Ibid.*

---

4. *On the safety-valve of the right ventricle of the heart of Man; and on the gradations of the same apparatus in Mammalia and Birds.* By J. W. King, Esq.*—"In this paper additional evidence is given by the author in corroboration of the principles which he had announced in a former communication, which was read to the Royal Society in May 1835, on the influence of the tricuspid valve of the heart on the circulation of the blood. His object is to demonstrate that the tricuspid valve in man occasionally serves the purpose of a safety-valve, being constructed so as to allow of the reflux of the blood from the ventricle into the auricle, during the varying states of distension to which the right cavities of the heart are at times subjected: that a similar function is maintained in the great number of animals possessing a double circulation, and also that in the different orders of these animals the structure of this valve is expressly adapted to the production of an effect of this kind, in various degrees, corresponding with the respective characters and habits of each tribe. He is thus led to conclude that the function which the tricuspid valve exercises exhibits, in the extent of its developement, a regular gradation, when followed throughout the different orders of Mammalia and Birds: and that it extends even to some Reptiles."—*Ibid.*

---

5. *On the brain of the Negro, compared with that of the European and the Ourang-Outang.* By Frederick Tiedemann, M. D.†—"It has long been the prevailing opinion among naturalists that the Negro race is inferior, both in organization and intellectual powers, to the European; and that, in all the points of difference, it exhibits an approach to the Monkey tribes. The object of the present paper is to institute a rigid inquiry into the validity of this opinion. The author has, for this purpose, examined an immense number of brains of persons of different sexes, of various ages, and belonging to different varieties of the human race, both by ascertaining their exact weight, and also by accurate measurement of the capacity of the cavity of the cranium; and has arrived at the following conclusions. The weight of the brain of an adult male European varies from 3lb. 3oz. to 4lb. 11oz. troy weight: that of the female weighs, on an average, from 4 to 8 oz. less than that of the male. The brain usually attains its full dimen-

---

* Communicated by Thomas Bell, Esq. [Read before the Royal Society.]

† Read before the Royal Society.

sions at the age of seven or eight; and decreases in size in old age. At the time of birth, the brain bears a larger proportion to the size of the body than at any subsequent period of life, being then as one sixth of the total weight: at two years of age it is one fourteenth; at three, one eighteenth; at fifteen, one twenty-fourth; and in the adult period, that is, from the age of twenty to that of seventy, it is generally within the limits of one thirty-fifth and one forty-fifth. In the case of adults, however, this proportion is much regulated by the condition of the body as to corpulence; being in thin persons from one twenty-second to one twenty-seventh, and in fat persons often only one fiftieth, or even one hundredth of the total weight of the body. The brain has been found to be particularly large in some individuals possessed of extraordinary mental capacity. No perceptible difference exists either in the average weight or the average size of the brain of the Negro and of the European: and the nerves are not larger, relatively to the size of the brain, in the former than in the latter. In the external form of the brain of the Negro a very slight difference only can be traced from that of the European; but there is absolutely no difference whatsoever in its internal structure, nor does the Negro brain exhibit any greater resemblance to that of the ourang-outang than the brain of the European, excepting perhaps, in the more symmetrical disposition of its convolutions.

"Many of the results which the author has thus deduced from his researches are at variance with the received opinions relative to the presumed inferiority of the Negro structure, both in the conformation and relative dimensions of the brain; and he ascribes the erroneous notions which have been hitherto entertained on these subjects chiefly to prejudice created by the circumstance that the facial angle in the Negro, is smaller than in the European, and consequently makes, in this respect, an approach to the ape, in which it is still farther diminished. The author denies that there is any innate difference in the intellectual faculties of these two varieties of the human race; and maintains that the apparent inferiority of the Negro is altogether the result of the demoralizing influence of slavery, and of the long-continued oppression and cruelty which have been exercised towards this unhappy portion of mankind by their more early civilized, and consequently more successful competitors for the dominion of the world."—*Ibid.*

---

6. *The Factory system and Factory statistics.*—In the present short notice of the influence of the factories on health, we do not intend to discuss the whole question, but simply to exhibit a few of the facts.

A slight sketch of the history of factory labour may not be unacceptable. While it shows the unrelenting inhumanity of avarice, it displays the difficulties with which philanthropy and benevolence must struggle, before they can effect their noble ends. The system of employing children as factory laborers was the result of Arkwright's inventions. His looms took manufacturers out of the cottages and farmhouses, and assembled them in the counties of Derbyshire, Nottinghamshire, and more particularly in Lancashire, where the machinery was used in large factories, built on the sides of streams capable of turning the water-wheel. There was an immediate demand for hands, and children were found to be best adapted for the work. The custom instantly sprang up of procuring apprentices from the different parish workhouses of London, Birmingham, and elsewhere. Many thousands of children, between the ages of seven and of fourteen years, were carted to the North. The horrors of this apprentice-system appear to have been dreadful. But it is past, and we need not rake them up again. But one fact may be stated. An agreement was made between a London parish and a Lancashire manufacturer, stipulating that, with every twenty sound children, one idiot should be taken!

The toil, the scourging, the crowding, the filth of the receptacles for the apprentices, reached such a height, that these outrages on nature were taken in hand by Nature herself, and pestilential fevers became alarmingly destructive. The public was roused, and a board of health was instituted at Manchester, and made its report in 1796. That report was highly damnatory of the factory system.

In 1802, the late Sir Robert Peel introduced and carried his first bill for the Relief of the Apprentices. It did relieve them, but steam being now applied to manufactures, the site of factories was removed from the sides of streams to towns and populous districts, where labour could be readily obtained, and where apprentices were an useless incumbrance. The bill afforded no protection for the children now employed, and, in 1815, Sir Robert proposed a second measure, which was not carried till the session of 1819. Sir Robert had limited the hours of actual labor to eleven, for all persons between the ages of nine and sixteen. The commons would not hear of eleven hours as an ultimatum, and extended the number to twelve.

The actual nature of the factory-labor was as follows:—

It was in nearly all the cotton-mills of Lancashire and its neighborhood, excepting Saturday, from thirteen to sixteen hours a day, inclusive of one hour, or less, *nominally* allowed for dinner. Many of those subjected to such labor were children of nine, eight, seven, and six years of age, and previously to the stirring of the investigation, under six, and, in some instances, *under five!* The children continued constantly at work so long as the machinery was in motion, during which time they were not permitted to sit down or to leave the factory. They often complained (naturally enough, our readers will think) of fatigue, and aching limbs; in this state of exhaustion, towards the close of the day, they were beaten by the spinners, or overlookers, or even by their own parents, that blows might supply the deficiency of srength. In most cotton-factories, during the greater part, and often the whole of the time *nominally* allotted for dinner, the children were occupied in cleaning the machinery; no time was allowed for the breakfast, or afternoon meals, which were snatched in mouthfuls during the progress of uninterrupted labor; the refreshments not unfrequently remaining untouched till it became cold, and covered with dust and dirt from the cotton-flyings. It appeared, moreover, that the temperature in many cotton-mills was from 75° to 80°, in others from 80° to 85°, and occasionally as high as 90°.

Such was the state of things when, in 1819, the twelve hours' Bill was carried. After the lapse of a few years, Sir John Hobhouse introduced an eleven hours' Bill, which was burked. He obtained, however, an act in 1825, which limited the labor of persons under sixteen to sixty-nine hours in the week, twelve on five days and nine on Saturdays. But his bill comprised the cotton-factories only. In 1831, it was somewhat improved by the prohibition of night work for all under twenty-one, and by the advance of the ages entitled to protection from sixteen to eighteen years. It is obvious that while something had been gained, humanity had yet much to effect, before she could be satisfied. Mr. Sadler, a very eloquent and a very earnest advocate of the cause of the factory-child, first appealed to the country in 1831, and brought in his bill in the ensuing session. The measure was cushioned, and Mr. Sadler never was in parliament again. A committee, which was appointed on the introduction of the bill, elicited a mass of evidence, which attracted the attention, and greatly excited the feelings of the public. A commission was now appointed by the crown, and the commissioners, four of them medical, were directed to investigate personally and systematically the condition of the children. Their report was presented to the house of commons in July, 1833. In March of that year, Lord Ashley had moved the first reading of a bill, resembling in all essential particulars, that of Mr. Sadler. It sought to restrict the labor to a period of ten hours, for all ages from nine to eighteen. The second reading of the bill was postponed until after the presentment of the report, which, as we observed, was in July. The report, in question, recommended eight hours as the ultimatum of labor for young children, and on that recommendation was based the law which is now in operation. The law in question, Lord Ashley's proposition being superseded, enacts :—

A period of eight hours for all under thirteen, and one of twelve for those between thirteen and eighteen. By a clause, it was provided that the act should come into operation by parts, each in succession. At the end of six months, children under eleven were to enjoy the benefit of the eight hours' limitation ; at the end of eighteen, all under twelve ; and at the end of thirty, all under thirteen. Subsequently, the ministry endeavored to vitiate the act, but met with a defeat, and it is at present the law of the land. There is every reason, however, for believing that a ten hours' bill for all ages will at no great distance of time be introduced, and as medical evidence has throughout been properly depended on as the basis of any legislative enactments, we have laid before our readers this sketch of the history of the various measures that have been attempted or adopted.

It only remains for us to notice some of the facts adduced, to show the actual working of the factory system.

Dr. Ray, in a pamphlet, entitled 'The Moral and Physical Condition of the Working Classes employed in the Cotton Manufacture in Manchester,' after presenting an elaborate survey of the streets, and the houses inhabited by the operatives—and after painting in the darkest colors the imperfect ventilation and the filth of all, introduces us to the person and the family of a cotton-spinner. Let us accompany the

Doctor down a lane containing 'heaps of refuse, deep ruts, stagnant pools, ordure, &c.,' and opening the door of what is facetiously called a house, let us gaze upon the inmates :—

"The population employed in the cotton-factories,' continues the Doctor, 'rises at five o'clock in the morning; works in the mills from six till eight o'clock, and returns home for half an hour or forty minutes, to breakfast. This meal generally consists of tea or coffee, with a little bread. Oatmeal porridge is sometimes, but of late rarely, used, and chiefly by the men; but the stimulus of tea is preferred, and especially by the women. The tea is almost always of a bad, and sometimes of a deleterious quality; the infusion is weak, and little or no milk is added. The operatives return to the mills and workshops until twelve o'clock, when an hour is allowed for dinner.

"Amongst those who obtain the lower rates of wages, this meal generally consists of boiled potatoes. Themess of potatoes is put into one large dish; melted lard and butter are poured upon them, and a few pieces of fried fat bacon, are sometimes mingled with them, and but seldom a little meat. Those who obtain better wages, or families whose aggregate income is larger, add a greater proportion of animal food to this meal, at least three times in the week; but the quantity consumed by the laboring population is not great. The family sits round the table, and each rapidly appropriates his portion on a plate, or, they all plunge their spoons into the dish, and with an animal eagerness satisfy the cravings of their appetite. At the expiration of the hour, they are all again employed in the workshops or mills, where they generally agian indulge in the use of tea, often mingled with spirits, accompanied by a little bread. Oatmeal or potatoes are, however, taken by some, a second time in the evening." 420.

During twelve hours of the day, the operative drudges, in a heated atmosphere, watching the movements, and assisting the operations of a steam engine. His toil is incessant, and rational relaxation he can have none, because, when the hours of work are over, bodily fatigue precludes every thing save indulgence in spirits, in the grossest sensuality, or in the indolence of mere exhaustion. What wonder then, that the factory laborers have become as a class degenerate in body—debased in mind—a grovelling, gin-drinking, demoralized set, whose animal vices are painted in the most revolting hues by those who have narrowly watched their habits. We have not room for the statements of Dr. Kay and of Mr. Gregg on this head, nor do they so much concern us, as the merely physical effects of labor.

It appears that the whole number of persons employed in cotton-factories in the year 1835, was 220,134, four-times as many as in 1818. Of that number, 94,287 were between the ages of eight and eighteen years—and 125,877, above the latter age.

It is singular to observe the effects of the improvements in machinery on the labor of the operatives. It will be remembered that in 1819, the legislature declared that twelve hours of mill-work were sufficient. The following calculation serves to exhibit the amount of labor undergone when that enactment was carried.

"A Table showing the distance over which a piecer had to travel, in following a pair of mules spinning cotton-yarn of No. 40, in the year 1815.

| The spinner put up 820 stretches daily, on each of two mules, 12 yards long each, and was attended by three persons. | Number of stretches daily. | Number of yards from mule to mule. | No. of yards comprising the piecers work along each mule, over which he must walk each stretch. | Total No. of yards. | Distance in miles. |
|---|---|---|---|---|---|
| | 1640 | 5 | 4 | 14,760 | 8 and a fraction. |

"The operation of the bill would appear to have done any thing but diminish the spinner's toil. The ingenuity of cupidity travelled faster than the legislation of humanity, as the next statement will demonstrate.

"A Table showing the distance over which a child must walk, in following a pair of mules spinning cotton-yarn of No. 40, at Manchester, in 1832.

| The spinner puts up daily 2,200 stretches on each of two mules | Number of stretches daily. | Number of yards from mule to mule. | Average No. of yds. which a child walks along each mule per stretch. | Total No. of yards daily. | Distance in miles. |
|---|---|---|---|---|---|
| | 4400 | 5 | 3 | 35,200 | 20.' |

At Bolton-le-Moors, the wheels would appear to revolve with still more astonishing rapidity, and it is estimated that a piecer, in attending a pair of mules spinning cotton-yarn, must there travel daily twenty-five miles! All this is done in a heated and impure atmosphere, by persons indifferently fed—an irksome daily drudgery, performed under the stimulus of absolute necessity. That the lower classes in a manufacturing country, or indeed in any, must either work or starve is true, and an exaggerated and preposterous compassion may be entertained on their behalf. But where the numbers of the laborers are such that the competition among them resulting from necessity, disables them from defending themselves against the exactions and tyranny of capital, both humanity and policy demand the interference of the nation, and the effectual protection of parliament.

If such reasoning is applicable to the case of adults, how much more forcibly must it apply to children—to those helpless creatures consigned by inhuman parents to almost as inhuman overlookers—who are made to taste the most sickening toil and care at the only age which nature has intended to be free from them. We cannot effect the Utopian project of releasing the poor man's child from labor, but we can and we ought to make that consistent with the powers of childhood. As medical philosophers we are called on to determine what those powers are, and what amount of work is consistent with the integrity of the infantile constitution. As medical practitioners we have been summoned to report, and probably we shall be summoned again, on the actual results of the existing system. The evidence that we have given has not always been calculated to exhibit either our candor, our science, or our good feelings, and we may be sure that the intelligent portion of the public both remarks and remembers these disadvantageous exhibitions.

It seems that the tendency of the factory system is to substitute more and more exclusively, the labor of the young for that of the adult.—There are many apparently good reasons for this, on which, however, we need not insist, as the fact is all we want. The consequence is, that the old are thrown on the young for support, the reverse of the order of nature. A sad picture has been drawn by too many observers of the subsistence of parents on offspring. That, however, is a consideration which belongs to the moral, rather than the medical philosopher, and we dismiss it. It concerns us more to hear that—

"The number of operatives above the age of forty is incredibly small. We must refer our readers to a curious but authentic document, arranged about the year 1831, during a great 'turn out,' from forty-two mills in Mosley, Ashton, Staley-Bridge, and Dukinfield, of 1665 persons, whose ages ranged from fifteen to sixty. Of these, 1584 were below forty-five; *three* only had attained a period between fifty-five and sixty; and not more than fifty-one between forty-five and fifty were counted, as fit for work! Mr. M'Nish, a witness entitled to the utmost credit, even by the admission of the commissioners, and in truth, as every one must perceive upon reading his evidence, a man of singular sagacity, deposed, in the year 1832, that, by actual enumeration of 1600 men in the factories of Renfrew and Lanark, he ascertained that no more than ten had reached forty-five years of age, and these, he added, were retained by the special indulgence and humanity of their masters. The spinners at that period are so broken down that they cannot produce the required quantity. 'Their eye-sight fails,' and then they are turned off, and younger men employed."

We now quit this subject. We have drawn the attention of our readers to it, because it is evidently still unsettled, and will again occupy the attention of the legislature. Medical evidence may once more be requisite, and it is well for the profession to be put in possession of the general facts, which will enable it to form a more comprehensive and more accurate estimate, of the various bearings of the question at issue. Independently of this, which may be said to be in some measure a personal consideration, it is daily becoming more indispensable for the medical practitioner to be well informed, on matters which affect the health of large sections of the nation. His opinions are frequently publicly asked, and if inconsiderately or ignorantly expressed, they redound to his own disgrace, and to the shame of the profession."

[*Med. Chir. Rev.*

—

7. *Special Anatomy.—On the Elementary Structure of Muscular Fibre of Animal and Organic Life.*—FREDERICK SKEY, Esq. has presented to the Royal Society an interesting memoir on this subject.

The author concludes, from his microscopic examinations of the structure of muscular fibres, that those subservient to the functions of animal life have, in man, an average diameter of one 400dth of an inch, and are surrounded by transverse circular striæ, varying in thickness, and in the number contained in a given space. He describes these striæ as constituted by actual elevations on the surface of the fibre, with intermediate depressions, considerably narrower than the diameter of a globule of the blood. Each of these muscular fibres, of which the diameter is one 400dth of an inch, is divisible into bands of fibrillæ, each of which is again subdivisible into about 100 tubular filaments, arranged parallel to one another, in a longitudinal direction, around the axis of the tubular fibre which they compose, and which contains in its centre a soluble gluten. The partial separation of the fibrillæ gives rise to the appearance of broken or interrupted circular striæ, which are occasionally seen. The diameter of each filament is one 16,000dth of an inch, or about a third of that of a globule of the blood. On the other hand, the muscles of organic life are composed, not of fibres similar to those above described, but of filaments only; these filaments being interwoven with each other in irregularly disposed lines of various thickness; having for the most part a longitudinal direction, but forming a kind of untraceable net-work. They are readily distinguishable from tendinous fibres, by the filaments of the latter being uniform in their size, and pursuing individually one unvarying course, in lines parallel to each other. The fibres of the heart appear to possess a somewhat compound character of texture. The muscles of the pharynx exhibit the character of animal life, while those of the œsophagus, the stomach, the intestines, and the arterial system, possess that of inorganic life. The determination of the exact nature of the muscular fibres of the iris presented considerable difficulties, which the author has not yet been able satisfactorily to overcome.—*Transactions of the Royal Society, for* 1836.—*American Journal of the Medical Sciences.*

—

8. *On the Connexion of the anterior columns of the Spinal Cord with the Cerebellum.* By Samuel Solly, Esq.—The exact line of demarcation between the tracts of nervous matter, subservient to motion and to sensation, which compose the spinal cord, has not yet been clearly determined. The proofs which exist of a power residing in the cerebellum which regulates and controls the actions of muscles, would lead us to suppose that the fibres of the motor nerves are continuous with those of the cerebellum; but hitherto no observations have been made which prove the existence of this connexion; and it is the object of the author, in this paper, to establish, by a more careful examination of the anatomical structure of this part of the nervous system, such continuity of fibres between the anterior columns of the spinal cord and the cerebellum. The corpora pyramidalia have been hitherto considered as formed by the entire mass of the anterior, or motor columns of the spinal cord; but the author shows that not more than one half of the anterior columns enters into the composition of these bodies; and that another portion, which he terms the *antero-lateral* column, when traced on each side in its progress upwards, is found to cross the cord below the corpora olivaria, forming, after mutual decussation, the surface of the corpora restiformia; and ultimately being continuous with the cerebellum. These fibres are particularly distinct in the medulla oblongata of the sheep and of the horse. The author conceives that the office of the antero-lateral columns is to minister to the involuntary, as well as to the voluntary movements; that the facial nerve arises from both the voluntary and involuntary tracts; and that the pneumogastric nerve arises both from the involuntary and the sensory tracts.—*Ibid.*

---

9. *Functions of the 5th, 6th and 7th pairs of Nerves.*—The *Revue Medicale*, for April, 1836, contains an interesting case of paralysis of the face, communicated by Dr. L. T. Desplanches, illustrative of the functions of the 5th, 6th and 7th pairs of cerebral nerves, and confirming the prevalent opinion, that the 5th presides over the sensibility, and the seventh over the mobility of the face. The author says he has seen eight cases of the same affection in which the anatomical lesions illustrate the same fact. In the case just alluded to there was paralysis of the external rectus muscle of the eye, caused by pressure upon the 6th pair of nerves, by the opthalmic vein, which was extremely dilated.

---

10. *On the Function of the Medulla Oblongata and Medulla Spinalis, and on the Excito-motory System of Nerves.*—Dr. Marshall Hall communicated to the Royal Society, at their meeting on the 2nd of March last, a memoir on this subject, the object of which is to unfold what he calls a great principle in physiology, namely, that of the special function, and the physiological and pathological action and reactions of the true spinal marrow, and of the excito-motory nerves. The two experiments which he regards as affording the type of those physiological phenomena and pathological conditions, which are the direct effects of causes acting in the spinal marrow, or in the course of the motor nerves are the following:—1. If a muscular nerve be stimulated, either mechanically by the forceps, or by means of galvanism passed transversely across its fibres, the muscle or muscles to which it is distributed are excited to contract.—2. The same result is obtained when the spinal marrow is subjected to the agency of a mechanical galvanic stimulus. The following experiment, on the other hand, presents the type of all the actions of the reflex function of the spinal marrow, and of the excito-motory system of nerves, and of an exclusive series of physiological and pathological phenomena:—If in a turtle, from which the head and the sternum have been removed, we lay bare the sixth or seventh intercostal nerve, and stimulate it either by means of the forceps or galvanism, both the anterior and posterior fins, with the tail, are immediately moved with energy. Hence the author infers the existence,—1st, of a true spinal marrow, physiologically distinct from the chord of intra-spinal nerves; secondly, of a system of excito-motory nerves, physiologically distinct from the sentient and voluntary nerves; and, 3dly, of currents of nervous influence, incident, upwards, downwards, and reflex with regard to the spinal marrow.

The author illustrates his peculiar views by several experiments and pathological observations, which appear to him to show that muscular movements may occur, under circumstances implying the cessation of sensation, volition, and every other function of the brain; and that these phenomena are explicable only on the hypothesis that impressions made on a certain set of nerves, which he terms *excito-motory*, are conveyed to a particular portion of the spinal marrow belonging to that system, and are thence reflected, by means of certain motor nerves, upon certain sets of muscles, inducing certain actions. The same actions may also be the result of impressions made directly either on the spinal marrow or on the motor nerves. He accordingly considers that the whole nervous system may be divided into,—1st, the *cerebral*, or the sentient and voluntary; 2ndly, the *true spinal*, or the excitor and motor; and 3dly, the *ganglionic*, or the nutrient, the secretory, &c. The excito-motory system presides over ingestion and exclusion, retention and egestion, and over the orifices and sphincters of the animal frame; it is therefore the nervous system of respiration, deglutition, &c. and the source of tone in the whole muscular system. The true spinal system is the seat or nervous agent of the appetites and passions, but is also susceptible of modification by volition. This theory he proceeds to apply to the explanation of several phenomena relating to the motions of the eye-lids, pharynx, cardia, larynx, muscles of inspiration, sphincter ani, expulsors of the feces and semen, to the tone of the muscular system generally, and to actions resulting from the passions. Lastly, he considers its application to various diseased states of the same functions, as manifested in cynic spasm, vomiting, asthma, tenesmus, strangury, crowing inspiration, convulsions, epilepsy, tetanus, hydrophobia, and paralysis.—*Proceedings of the Royal Society, for* 1836.—*American Journal of the Medical Sciences.*

---

11. *On the importance of Percussion in the Diagnosis of Ascites and Encysted Ovarian Dropsy.*—M. Rostan considers the signs afforded by percussion of the utmost importance in the diagnosis of ascites and encysted ovarian dropsy, where the diagnosis is difficult. When we strike the belly of a patient laboring under ascites, where the effusion has not gone so far as violently to distend the abdominal parietes, if we proceed from the inferior to the superior part of the abdomen, we shall at first have a flat sound, while, at the most prominent part—that is, about the umbilical or epigastric regions—we shall perceive a clear sound, like to that which we obtain on striking a bladder full of air. In encysted ovarian dropsy the reverse is the case; that is, the sound is clear at the inferior part, and dull at the superior. These differences are produced by the different relations which fluid effused into the peritoneum, and an ovarian cyst, have to the intestines. The dullness which we find at the inferior part of the abdomen in ascites, is the result of the accumulation of the effused fluid, which gravitates to the most dependent part of the peritoneal cavity, and while the collection of fluid augments there, the intestines become displaced; and it is important to know of this displacement, because it is it which accounts for the clear sound in the superior regions. In fact, this sound is the result of the less specific gravity of the gases contained in the intestinal cavity, in virtue of which the intestines are raised upwards, as the fluid on which they float accumulates below. The state of the parts, in encysted ovarian dropsy, is not at all the same; for in ascites the fluid is freely spread out within the peritoneum, while, in the other case, it is shut up in a more or less resisting bag, which gradually increases in size by the accumulation of serous fluid in its interior, and, as it becomes displaced, presses on the intestines of the side opposite to that on which it developes itself, and at last ends by occupying a greater or smaller space in the abdomen. This case is very different from the first; here the intestine does not float in the accumulated fluid, as it is separated from it by a wall of membrane; and therefore, instead of being raised upwards, it will be kept and pressed against the inferior part of the abdominal parietes; and thus, in this case, we find its dull sound high up, corresponding to the cyst, and its clear one low down, where the intestines are to be found distended with gas. In the generality of cases these signs are sufficient for the practitioner; but they may not be sufficient under certain circumstances which are not common, and which may be reduced to the following:—1st. It may happen, in either case of dropsy, that the intestine is not distended with gas; in that case, the signs which have been just pointed out become useless; but this state is only transitory, as the formation of gas takes place during digestion. In such a case the examination need only be deferred a few hours. 2nd. We may meet with a case of ascites, in which the walls of the abdomen are so much distended, that the mesentary is not long enough to allow the intestine to float freely in the fluid. In that case you must percuss the patient, making him vary his posture. 3d. We may suppose that a cyst, in its development, occupies a place between the intestines and the posterior wall of the abdomen, and is situated under the mesentery; and that, in augmenting its size, it forces the mesentery along with the intestines towards the superior part of the abdomen. In this case, we must confess, there will be a great source of error, because percussion will give the signs belonging to ascites. It is in cases like these that we feel the importance of not confining ourselves to one sign, but of accurately scrutinizing every symptom that can throw light on the diagnosis.—*La Presse Medicale.*—*Ibid.*

—

12. *Clinical Researches on the influence of certain Medicines on the functions of the Heart.* By H. C. Lombard.—1. Assafœtida. M. L. states this to possess remarkable properties in combating the irregularity of the functions of the heart. Employed *externally*, in the form of plaster, it succeeds in alleviating palpitations which have resisted a great variety of medicines. He has almost constantly obtained some alleviation in a great number of cases. Irregular contractions of the ventricles, occurring in persons affected with disease of the heart, are modified; and it likewise succeeds in those cases which may be considered only nervous. The following is the formula used by him:—Assafœtida, 2 ounces: gum ammoniac, one scruple; turpentine, 6 drops; yellow wax, a sufficient quantity. Employed *internally*, he has found it likewise lessen and render regular the movements of the heart. In very small doses, it lessens the palpitations, and produces a remarkable calm; and he considers it a very valuable remedy in nearly all diseases of this organ.

2. *Camphor.* This medicine, given internally, in variable doses, from three to twelve grains in the day, acts in a special manner upon the heart. Among persons affected with hypertrophy, with dilatation of the cavities, the nervous influence is often insufficient to produce regular and complete contractions, and hence often tumultuous action. This state he has found can be modified by camphor, and he has seen the most tumultuous ventricular contiactions become regular and perfectly isochronous after the administration of a few grains. He is not able to decide whether it acts as a stimulant or a sedative.

3. *Digitalis.* M. Lombard believes the want of uniformity in the sedative action of this medicine upon the functions of the heart, depends upon the four following circumstances:—1. The state of the stomach: 2, the mode of life of the patient; 3, the doses given; 4, the mode of administration. Sometimes, owing to an irritable state of the stomach, the exhibition of digitalis induces vomiting; and, if this continues after the cessation of the medicine, we must not have recourse to antiphlogistic measures, but to antispasmodics; such as the subnirate of bismuth, oxide of zinc, and effervescing draughts. The mode of administering digitalis is one of the most important points in its therapeutical history. The infusion is the preparation which produces most promptly symptoms of saturation. In the form of powder it rarely produces vomiting, except when the doses are large and frequently repeated. The best medicines for obviating or allaying these symptoms of saturation are calcined magnesia, subnirate of bismuth, subcarbonate of iron, or oxyde of zinc. M. L. considers the subcarbonate of iron as the best, and thinks he can attribute to its use the absence of banefnl results among his patients who took digitalis daily for many months.

4. *Polygala Senega.* The therapeutical action of this medicine is little known. M. Lombard considers it one of the most precious which the materia medica possesses. Administered in the form of extract or infusion, he has found it lower the circulation, and especially regulate the ventricular contractions. The dose employed varied between twelve and twenty-four grains in the course of the day. The infusion, prepared with one drachm to four ounces of water, has been often administered in the same time.—*British and For. Med. Rev.*, from *Bulletin Gen. de Therap.* Nov. 1836.—*Ibid.*

---

13. *Physiological effects of Tannin.*—M. Cavarra has lately made some experiments on this subject. He administered the tannin, in its pure state, to dogs, to the extent of twelve grains, and himself took for three days running three pills, each of 2 1-2 grains. In both cases obstinate constipation was the only effect produced. He killed a dog to whom he had given it, and found the mucous surface of the intestines dry, and the fecal matter hard and collected in the colon, the membrane of which, when examined with a magnifying glass, displayed a remarkable closing of the pores and villi. M. C. has successfully treated with pure tannin six cases of diarrhœa, twenty-three cases of leucorrhœa, and five of pulmonary catarrh, with some cases of hæmoptysis, hemorrhage from the rectum and vagina, and a few of gonorrhœa. He administered it either in the form of pills or of a solution.—*Revue Therapeutique.—Ibid.*

---

14. *Therapeutic applications of Indigo.*—This substance was first employed as a therapeutic agent in the treatment of epilepsy, by Lenhossek, and afterwards by Grossheim and others. Its efficacy was afterwards tried by Ideler, a Prussian physician; and among twenty-six patients, in whom indigo was experimentally tried, six individuals recovered completely; three were dismissed cured, who had after intervals of from eight to twelve months a relapse, under the operation of causes which might have induced epilepsy; of eleven patients, the condition underwent an essential improvement; and in six individuals no change took place. At first, the patients were wont frequently, though without effect, to vomit; after some days this ceased, and in its place there took place diarrhœa, which at first caused from six to eight motions daily, and was occasionally accompanied with moderate colicky pain, but afterwards moved the bowels only two or three times daily, but with fluid motions, and continued so long as the indigo was used, but without impairing the appetite or digestion. The curative reaction of the nervous system upon the agent was principally indicated by this circumstance, that the epileptic symptoms in the first period returned more frequently, and attained a higher degree of intensity, but afterwards became less frequent, milder, and at length entirely disappeared.

Most usually the indigo was exhibited in the form of electuary, with a proportion of the aromatic powder, because, alone, it is very disagreeable to the patient. At first it was administered in the dose of one scruple; this was quickly increased to a drachm and more, so that daily from one half an ounce to one ounce might be used for a series of months without difficulty.

In a paper in Grafe and Walther's Journal, entitled Contributions to Casnistics, by D. Moritz Strahl, of Berlin, are some observations on the operation of the same remedy in spasmodic diseases. In the trials made by Dr. Strahl with this agent, in ten cases of inveterate epilepsy, in which it was given in progressively increasing doses, of from one scruple three times a day, to half an ounce daily for the space of ten weeks, it produced not the smallest effect. During its employment the stools became blue, and the urine assumed a dark green color. Excepting slight inconvenience of the stomach, no operation of the remedy upon the organism could in general be observed. On the other hand, indigo, in four hysterical females, one of whom was already in the age of decrepitude, evinced the presence of very remarkable phenomena. In all, after about two drachms daily had been taken, violent pain in the region of the kidneys, like colic, took place; the urine assumed a deeper intensity of coloring than in male patients, and at the bottom of the vessel was observed no trifling quantity of fine indigo powder. The intense renal pain continued for four days, and at length subsided under the continued employment of an oily emulsion. In one case only did there ensue a remission of the spasms, and the patient was not entirely well three months after the cure was completed. The operation of the indigo, further, on the womb, was very remarkable, since, in two cases, an amenorrhœa was radically cured, while the spasms were throughout undiminished. In two cases of St. Vitus's dance, in a boy of 12 and a girl of 9 years, the indigo was throughout unavailing.

The different clinical trials made with indigo by Dr. Roth, furnished the following results. In epileptic cases, the remedy evinces almost always the same immediate operation; but its subsequent consequences are regulated by the degree of vitality of the nervous system of the patients, and the kind and duration of the epilepsy. These effects are beneficial in all idiopathic epilepsies, curative in those of this class which have not been of long continuance; and in very chronic idiopathic epilepsies, indigo diminishes the violence and the frequency of the paroxysms. Of symptomatic epilepsies, only a few are alleviated by the use of indigo, none are cured.—*Edinburgh Med. & Surg. Jour.*, from *Neue Wissenchaftliche Annalen.—Ibid.*

---

15. *Physiological operation of Indigo.*—In almost all patients, the use of indigo is succeeded first by squeamishness and vomiting, tho' the substance itself be tasteless and inodorous. The violence of the emetic efforts appears to be regulated by the individual irritability of the gastric nerves of the patients. Females vomit more readily than males. The vomiting is at first continuous, that is, during the continued use of the agent, and often so violent that the indigo must be given up; but after several days it ceases. It has otherwise the peculiarity that the contraction of the abdominal muscles and the diaphragm is much less violent, and the debility is less considerable than after vomiting induced by other means. The contents of the stomach present nothing unusual, even in respect to taste, only they are of a very dark blue color, and the fluid is intimately mixed with the indigo, from which it may be inferred that the gastric juice contributes very much to the digestion of the indigo.

Diarrhœa, the second physiological effect of indigo, takes place in general first when the vomiting ceases; yet from this many patients remain altogether exempt. In general, diarrhœa, when once commenced, continues as long as the patients take the indigo, and increases in intensity during the continued use of the remedy. The motions are generally soft, semifluid, and of a dark blue-black color. The vomiting and diarrhœa are frequently accompanied with slight colicky pains in the stomach and bowels, which, however, may be so violent as to require the indigo to be given up. Those patients who are exempt from vomiting appear to be attacked with more violent colicky symptoms. By the continued diarrhœa there is formed a species of gastrosis (irritation of the mucous membrane of the stomach and bowels,) with loss of appetite, headache, and giddiness, and sometimes the sense of dazzling lights in the eyes.

The third physiological operation of indigo is seen in the urinery secretion. The urine assumes a dark violet color, deepest in the morning. On the quantity of the urine the agent seems to exercise no influence.

Dr. Roth did not observe coloration of the sweat. But it is remarkable, that one patient, after the use of indigo for several weeks, fell into slight convulsions, similar to those which ensue on the employment of the nitrate of strychnia.

The dose of indigo is regulated by the irritability of the stomach. It is best to begin with grains, and rise gradually to drachms, or even several ounces daily. Dr. Roth gives the preference to the form of electuary, with proportional additions of the aromatic, or Dover's powder, as correctives. In the formula employed in the Hospital of the *Charite*, at Berlin, half an ounce of powdered indigo, rubbed up with a few drops of water, is mixed with half a drachm of aromatic powder, and one ounce of simple syrup, and to be taken in divided doses in the course of the day. Many even take from a half to two ounces, twice and four times daily for the space of several months.

In what manner indigo operates, and to what class of medicines it belongs, is very difficult to determine, and certainly cannot be inferred from its constituent parts. Probably its active principle is seated in the peculiar coloring matter. Though in many respects the operation of indigo is similar to that of tartar emetic, yet this attacks more forcibly the energy of the organism. In all the patients after the use of the indigo the spasms were at first more frequent and more intense, but shorter in duration; but after some weeks their intensity was manifestly abated, and at length they entirely disappeared. All the patients cured by indigo labored under idiopathic epilepsy, that is, epilepsy without symptoms of organic lesion. Among those who were improved were several idiopathic and symptomatic cases. In one case of epilepsy, which ensued after a remarkable contusion of the head, after the employment of indigo, a moderately long intermission took place. A boy of 16 years of age, who had labored for eight years under St. Vitus's dance, and then was attacked with epileptic spasms, was cured of all the symptoms by the use of indigo for six weeks. Of twenty-six epileptic patients treated by means of indigo, there recovered four males and five females; three males and eight females were improved; and four males and two females remained uncured. In confirmation of the foregoing inferences, the author communicates the history of two cases, in which the treatment by means of indigo operated beneficially, after other means had been found unavailing.—*Ibid.*

---

16. *Observations on Jaundice; more particularly on that form of the disease which accompanies the diffused inflammation of the substance of the liver.* By Richard Bright, M. D.—The causes which generally give rise to jaundice will, perhaps, admit of the following classification:—

1. Congestion of blood in the liver. 2. Obstruction of bile in the biliary ducts, and more particularly in the larger ducts. 3. Chronic change in the structure of the liver. 4. Inflammatory action of the liver.

1. Congestion of blood takes place in the liver under various circumstances, of which the following are the most frequent:—Obstruction to the circulation of the chest, more particularly from valvular diseases, or dilatation of the heart; constipation, pregnancy; and certain conditions of the circulation, connected with remittent and other fevers.

2. Obstruction to the passage of the bile in the larger ducts takes place from biliary concretions; from malignant or other growths in the liver itself; or in glands about Glisson's capsule; from changes in the coats of the ducts themselves; from inflammation of the duodenum; or from induration of the pancreas.

3. The chronic changes in the structure of the liver are of various kinds; sometimes the result of simple inflammatory action of a somewhat acute character, imperfectly subdued; at other times, the result of a very slow and chronic action, affecting either the secreting portion or the cellular tissue; at others, the result of degeneration, or of malignant action taking place extensively throughout the organ.

4. The inflammatory action of the liver, which chiefly gives rise to jaundice, occurs in its substance, sometimes, if unchecked, going on to suppuration; but at other times producing a gradual change in the texture of the liver.

It is my intention, before bringing forward a few cases to illustrate the fourth class of causes to which I have referred, and which forms the chief object of the present communication, to state briefly some of the more prominent circumstances attending the other three classes; that, to a certain extent, we may have an opportunity of comparing, or of contrasting, their various symptoms, and the morbid changes by which they are marked.

When obstruction takes place to the circulation through the chest, but more particularly when the heart becomes gorged and over-distended with blood, we observe the countenance gradually assume a dingy aspect, in which the purple suffusion of carbonized blood is mingled with the yellow tint of a slight jaundice; the conjunctiva is more decidedly tinged; and if the disease continue long, the jaundice sometimes completely prevails over the purple tint; the urine becomes scanty, and high colored, throwing down the lateritious sediments; but the dejections are not obviously deficient in bile. In this condition, the primary disease has occasionally been overlooked; and this error has been confirmed, when, on examination in the right hypochondrium, the liver has been found enlarged, descending three or four inches below the margin of the ribs, and decidedly tender and painful on pressure. In these cases, the obvious embarrassment in the chest, and the peculiar distress and anxiety of countenance, will generally present themselves in conjunction with the faint and dingy color of the jaundice, as evidence of the original disease; and lead us to seek those stethoscopic signs, which will render still more obvious the nature of the obstructing cause.

Should death occur, it will probably have been preceded by the passage of blood, more or less freely, from the lungs or the intestines; and the examination of the body will demonstrate, that the liver has only partaken, with other organs, in the congested state of the venous system. The liver, under these circumstances, is sometimes found in a simple state of congestion throughout all portions of its tissue, to such a degree, as to give it a general dark color, and afford an abundant flow of blood when an incision is made; but at other times, when the congestion has been of longer duration, it presents that mottled appearance which has been correctly compared to the section of a nutmeg. In the early stages this appears to be simply a state of sanguineous congestion; but when it has continued long, some more fixed deposit seems to take place, and many of the acini assume a light yellow color, and a degree of firmness which is not found in the healthy organ. In connexion with this condition of the liver, the mesenteric veins will be found full of blood; the villous membrane of the intestines pretty generally of a deep red color, but varying in different portions; the internal surface of the stomach often suffused to such a degree as to suggest the idea that some deleterious substance has been administered; the pancreas of a purple or a leaden hue: and while thus the abdominal viscera are loaded with venous blood, the lungs are gorged, and blood is effused into them, in the form of apoplectic masses; and, in all probability, a certain quantity of serum, tinged with blood, and slightly colored with bile, will be discovered in the cavities.

The treatment of this form of jaundice resolves itself in those means which are calculated to relieve the original disease; but much assistance is to be derived from the local abstraction of blood; by cupping from the pit of the stomach; or by the application of leeches to the anus, which more directly unloads the vessels of the abdominal circulation.

The second class of causes upon which jaundice depends includes those cases in which, owing to diseased structure or vitiated secretion, some positive mechanical obstruction takes place, preventing the flow of bile from the larger ducts, and thus retaining it within the substance of the liver. In cases of this kind, we usually have a very vivid color displayed upon the skin; which takes place either suddenly, or by slower degrees, according to the precise nature of the obstructing cause; and which continues a longer or shorter time, likewise, according to that cause; either ceasing altogether, or continuing till death takes place, at no very distant period; or passing gradually into that dingy green color which, at first sight, impresses the eye almost like the countenance of the mulatto; and, in that sense, may not unfairly deserve the appellation of black jaundice. Amongst these mechanical causes, the two most frequent are, undoubtedly, biliary concretions, and malignant tubera.

The presence and even the passing of biliary calculi is by no means necessarily accompanied with jaundice; for as long as the cystic duct alone is obstructed, or the hepatic or common duct only partially blocked up, the fixed yellow color may show itself, although slight indications of jaundice may be present, and may recur frequently, passing away with surprising rapidity, so that almost daily changes may be observed. But, on the other hand, when the common duct is blocked up, the most brilliant jaundice often takes place, and the same in the more rare cases of the calculus being lodged in the hepatic duct. This color will occasionally be diffused most suddenly; and when this is the case, if the obstruction be not removed, a fatal result will sometimes speedily follow.

The evidences of biliary concretions, as deduced from the appearance of the jaundice, are therefore by no means determinate and certain; but the pain with which the disease is generally accompanied may be considered one, at least, of the most prominent symptoms which attend the passing of calculi, and assist in throwing light on the cause of jaundice. That pain is of two kinds,—a dull aching pain, which is constant, and an acute agonizing pain, which comes and goes in paroxysms. The severity of the pain is so extreme as to bring on a state of the greatest exhaustion, and reduce the pulse below the natural standard, both as to strength and frequency, or still more often, to render it rapid and weak, while the hands and the whole surface are bedewed with a cold perspiration. The urine becomes highly tinged with bile, and the stools of a pale drab color; but this often varies in the course of the disease. Vomiting, hiccup, and a frequent catching inspiration often accompany this form of disease, and the symptoms are aggravated or diminished as the paroxysms of pain advance or recede. Another very frequent symptom is to be found in the occurrence of rigors at somewhat irregular intervals; but sometimes returning periodically, almost with the exactness of an intermittent. This form of jaundice is very apt to return from time to time, either from the successive passing of calculi which are seldom solitary, or from the ineffectual attempts made to get rid of a large calculus.

Should death occur in this form of jaundice, the cause of the biliary obstruction is generally detected without difficulty, by finding concretions in some of the various forms they assume, blocking up partially or completely the passages by which the bile should escape; and very frequently we have an opportunity of explaining peculiarities which have occurred in the course of the disease, by the situation occupied by the calculus. It is by no means necessary that the calculus should be large, for we occasionally find the obstruction to have been complete, though the gall-stone has been of very moderate dimensions; and occasionally the calculus has passed, leaving the duct greatly distended, but still the constitution has not been able to rally from the effects of the disease. The liver, in this form of the disease, is found considerably loaded with bile, and the bile-ducts often distended, sometimes forming pouches in which bile is collected, showing a long-continued and frequently-returning tendency to obstruction.

The treatment of jaundice, under these circumstances, is to be directed to the removal of the temporary obstruction, and consists of such means as are likely to favor the passage of calculus. Opiates combined with purgatives, warm bath and assiduous fomentations, are amongst these means; but besides these, the use of mercurials and antimony are of great importance. The antimony, with a view of relaxing spasms, should be combined with the purgatives, wherever vomiting is not a very prominent and distressing symptom. The use of mercury is much more doubtful, and it ought not to be carried to any great extent; for if the obstruction be very obstinate, we run a great risk of doing mischief by over stimulating the liver, and shall probably add to the distress under which the patient labors, both by increasing the quantity of bile secreted, when there is means of carrying it off, and likewise by increasing the irritability and diminishing the powers of the system by the action of the remedy. In these cases, then, there is no doubt that injury is not unfrequently done by the administration of mercury; but this is still more liable to be the case in those instances of jaundice from mechanical obstruction, which arise from organic deposits.

The most frequent instances of organic deposit giving rise to jaundice are those in which malignant disease establishes itself in some of the complicated parts which lie in the neighborhood of Glisson's capsule—the small lobes of the liver, the glandular structures, the pyloric end of the stomach, the substance of the duodenum, or the pancreas.

Where any organic lesion of this kind takes place, the jaundice is generally less sudden in its appearance than in other cases, though I have known apparent exceptions to this rule,—I say apparent, because, in many cases, particularly amongst the poor, disease may have made a gradual but decided progress, without exciting attention, till the change of color has become very observable, and then it is supposed to have originated suddenly. It happens much more frequently that the countenance has gradually become suffused with bile, but at length the more decided jaundice has taken place, and this has gone on increasing in intensity for a time, after which the color has lost its brilliancy, and assumed the dark dusky green hue and squalid appearance, which is one of the worst symptoms. In this form of jaundice the urine becomes loaded with bile, till it assumes a color deeper than porter, but of a green tint; and the stools are of the lightest drab color, approaching to white. Now, the patient becomes drowsy, ecchymosis takes place in various parts, blood escapes from different surfaces, the frame emaciates, and a state of exhaustion, succeeded frequently by coma, closes the scene.

Malignant disease may, however, exist in the liver to a very great extent, without producing any marked or decided jaundice; for as long as it develops itself only near the surface, or in such parts as it does not make pressure on any large biliary duct, although slight effusion may result, the decided jaundice will not appear; but, when the malignant growths occupy parts external to the liver, but are so situated as to press upon the hepatic or the common duct, this system shows itself in its greatest intensity.

There are other symptoms, more particularly those connected with the evacuation from the bowels, which accompany the complete retention of the bile—occurring in cases where pressure is made on the common and perhaps the pancreatic duct—to which attention ought to be directed; though, as yet, no certain indications can be drawn from them. I refer to the evacuation of fatty matter, more or less mingled with the fæces. In several cases where the obstruction has been in the pancreas itself, a considerable quantity of that substance has been floating, and completely separated from the fæces; of which another instance, besides those I have already published, has lately occurred to me in the hospital. But in most cases of very obstinate jaundice, accompanied by complete obstruction, an unusual quantity of fat has been detected; and my friend, Mr. G. O. Rees, has kindly undertaken several analyses for me, with a view of ascertaining this fact; the result of which may, at some future time, be more fully stated.

The appearances presented by the liver, where organic mechanical obstruction has long existed, are generally those of the most marked accumulation of bile in all the ducts, behind the obstructing cause, whether they be the larger or the smaller, so that, in some cases, the whole liver assumes a deep olive-green color, or occasionally a still more vivid shade of green; the large ducts within the liver are distended to the size of the finger; the hepatic, cystic, and common ducts are of still greater dimensions; and the gall-bladder, containing several ounces of bile, projects far beyond the margin of the liver; the fundus forming a tumour, which has been perceptible to the touch, during life.

The other appearances are those which belong to the disease producing the obstruction, and to the effects of the jaundice on different organs. Amongst the former are tubera of various characters, but generally of hard and solid consistence, or softening towards their centre; enlarged and hardened glands; or a scirrhous state of the pancreas; and frequently a complete and permanent closing of the common ducts.

The treatment in these cases is necessarily little more than palliative. As long as the symptoms have not fully convinced us of the nature of the disease, we may be induced to employ mercurials cautiously; but where the disease is confirmed, mercury can be of little or no utility, and will generally be productive of very injurious consequences: it ought, therefore, to be avoided, except in the form of an occasional purgative, and whatever can allay irritation, support the tone of the stomach, and supply gentle nourishment to the system, will afford the best means of prolonging life, and diminishing the suffering of the patient. What power iodine, in any of its forms, may be capable of exerting over deposits of this kind, or how far it may be able to check the progress of their formation, is at present not sufficiently ascertained.

The third class of causes includes various chronic changes in the structure of the liver; and in these, many of the symptoms present a marked difference, when compared with those of the two foregoing classes. The color of the skin seldom partakes either of the purple tinge which attends thoracic obstruction, or of the deep yellow, or the dingy green, which are so frequently observed in cases of mechanical closing or obliteration of the life ducts. The change from the natural color is usually gradual and inconstant; and the yellow tinge of the conjunctiva often precedes, for some weeks, any more decided indication. In time, however, the bronzed appearance of the forehead, or the darkened areola of the eye, bespeak the approaching change; and a jaundice, bearing the lighter tints, from a sallow suffusion to a fainter or more decided lemon hue—still, however, liable to considerable fluctuation—establishes itself over the whole body.

The urine is usually scanty, and pretty deeply colored with bile, frequently depositing the pink sediments in abundance. The alvine evacuations seldom present that marked deficiency of bile which in some other cases is observable; on the contrary, they vary through the different shades of brown and yellow; and are often remarkable, rather for the unequal manner in which the bile is mingled, than for the absence of that secretion. The action of the bowels is generally irregular; and as the disease advances, evacuations of blood frequently take place. Ascites and anasarca usually follow quickly in the train of these diseases.

The more frequent cause which excites this form of jaundice is the excessive use of stimulating food and drink; inducing long-continued or frequently-repeated over-action of the liver, rarely amounting to an appreciable state of inflammation. It likewise arises as a sequel to more decided and even inflammatory attacks; and is sometimes the result of the diffused scirhrus propagating itself through the organ.

The appearances presented after death vary greatly; but, as regards the liver, are all, more or less, indicative of long-continued morbid action. The liver is sometimes increased in its size; but very frequently quite the contrary, the organ having evidently undergone contraction in the progress of the disease: indeed I have, in some cases, most distinctly traced its enlargement in the beginning of the attack, and its gradual diminution towards the more-confirmed stages of disorganization. Though the larger ducts are pervious, and a certain quantity of imperfect bile is found in the gall-bladder, yet the whole substance of the liver is frequently tinged by the bile retained within its smaller ducts. A general granular appearance exists throughout the liver, as if the acini were drawn into masses, surrounded by thickened cellular membrane: and if, without being injected, a portion of liver in this state is macerated in water for several weeks, the little granules assume the appearance of adipocire; and may be easily washed out by a stream of water, leaving a fine tissue of vessels and cellular membrane floating in the water. When the disease has gone further, the bands of cellular membrane are seen intersecting the structure, and forming more decided septa between the masses of acini. These are, the more common appearances, where, from frequent over-stimulation, gradual change in the structure of the liver has given rise to jaundice. Of some of the appearances produced by malignant deposits distributed generally throughout the organ, I shall take another opportunity of speaking.

Besides the changes in the liver, a great many others, affecting the organs of the abdomen and the peritoneum, might be mentioned; but that which is most frequent is the disease and ulceration of the lining membrane of the colon, which very generally occurs where the structure of the liver has gone into chronic degeneration; and in no case more frequently, than when the true fatty degeneration, which is seldom accompanied by jaundice, has taken place.

The treatment in this third class of jaundice is rather by the cautious adaptation of food, the avoiding of stimulus, and the long-continued employment of such medicines as restore and keep up the moderate action of the system, than by any powerful remedies. Much may be done in the early stages, if the patient will submit to regimen; but when serous effusions have taken place, and other symptoms of advanced disease have shewn themselves, little but temporary relief can be expected.

It will, however, I think, be found, that one of the most common causes of jaundice is what I have assumed as a fourth cause—a state of inflammatory action more or less generally pervading the substance of the liver; and it is highly probable, that in different cases different constituent portions of this intimate structure become more affected than the rest; but this is a point which requires stricter investigation than has hitherto been bestowed upon it. I consider this inflammatory state, in many respects, different from that very chronic action of which I have lately been speaking, and which may rather be called a state of long-continued irritation, than a state of inflammation. The causes from which it arises are frequently very different, and, besides irregularities of diet, include atmospheric exposure, the effects of external violence and injuries, the irritating effects of biliary concretions and of the retained bile, and perhaps the stimulating action of mercury.

The symptoms likewise differ in many respects from those of the other forms, though not separated by sharp and abrupt lines of demarcation; and the changes produced on the substance of the liver are decidedly different, affecting much more generally the secreting portion than the connecting cellular tissue; and probably involving the branches of the portal vein in preference to other parts.

The progress of inflammation, in these cases, varies so greatly in its intensity and in its rapidity, as to allow of a division being made into the more and less acute forms. It frequently comes on very insidiously, with symptoms and feelings of general constitutional derangement, depression of spirits, slow pulse, oppressed breathing, wandering abdominal pains, constipated bowels, and sometimes sickness of the stomach. In a day or two, the conjunctiva becomes tinged; and in a few days more, there is universal bright bilious suffusion of the skin. It is now found that the pulse is either accelerated, or sometimes still oppressed; and frequently, on pretty severe pressure about the region of the liver, some degree of tenderness is manifested; while in other cases, pressure produces little or no immediate suffering, but the pain comes on gradually a short time after the pressure has been made, and continues for hours or days. Cases of the less acute kind generally yield readily to treatment, if it be adopted early; and they form a large proportion of the cases of simple jaundice which present themselves in practice. In other cases, the inflammatory action is attended with much more severe symptoms, with considerable pyrexia, quick pulse, flushed countenance, and dry tongue, while a jaundice of the most intense color is diffused over the whole surface. The stools are, both in the more and less acute cases, of a light color; but less decidedly so, and subject to greater variations than when the obstruction is mechanical; and occasionally, after a few days, give little evidence of deficiency of bile. The urine is deeply tinged. When the disease assumes its more active and febrile form, those symptoms referable to the brain and nervous system, and which appear to depend upon the deleterious effects of bile circulating in the blood, are more intensely marked than in any other form of jaundice; and the tendency to hemorrhage sometimes comes on very early, and is excessive. In some cases, rigors, which assume the form of irregular intermittent paroxysms, form a prominent feature, as the disease advances; and then it often happens, though not always, that suppuration is established; and this may be going on to a great extent, while still the jaundice has rather decreased, or varied exceedingly in its intensity.

In cases of jaundice from inflammatory action, the condition of the liver after death differs according to the period at which the disease has proved fatal; but, in general, the size of the organ is not materially increased; though, on the contrary, it is not unfrequently perceptibly diminished. There is no accumulation of bile in the minute ducts, and the yellow tinge which pervades certain portions of the structure is scarcely more than other structures of the body have obtained from the bilious impregnation with which the blood is loaded, and bears no analogy to the dark green of the liver, loaded with bile from obstruction in the large ducts. On examining the gall-bladder, it is found to contain little bile, and sometimes scarcely a trace of that fluid is to be discovered coloring the mucus accumulated by the secretion of its lining membrane.

When the disease has terminated early in its course, the whole liver seems rather soft and flaccid; the surface appears variegated, of a light yellow, and dark red or purple, in patches; and certain portions project above the rest, which, when cut through, sometimes prove of a softer texture, and even to be undergoing a process of change or disorganization; and portions of the same kind are intermixed throughout the whole substance of the liver; while, at other times, the yellow portions are harder than the surrounding substance.

If the disease has not proved fatal at the early period, and while the jaundice is in its brilliant and intense form, but has gone on for some weeks, till the skin has assumed the light lemon-color tint which often bespeaks a very general disorganization of the liver, we find the structure extensively altered, and a great many of the acini apparently altogether incapable of receiving such a quantity of blood as is necessary for the secretion of the bile, or for giving the healthy color to the organ. They are then of a whitish-yellow color, and rather hard and contracted than enlarged; and these altered acini are seen in groups and clusters, which, on careful examination, will generally be found to follow the course of the divisions of the portal vessels, so as to be disposed around them like a sheath, which sometimes extends to the thickness of a quarter of an inch.

There is still another condition of the liver, apparently resulting from this diffused inflammation of its substance, and which occurs when the stage of suppuration has become gradually developed; we then find the liver pervaded by a multiplicity of abscesses, all of which seem tending to discharge themselves into branches of the portal vein, which then assumes a most unhealthy suppurative appearance along large portions of its course.

With regard to *treatment*, in cases where jaundice depends upon inflammatory action, it must always be decided antiphlogistic; but it is only where it presents itself under the more violent forms, that general bleeding need be employed. In other cases, cupping from the margin of the ribs, and—as soon as the bleeding is stopped—the assiduous application of poultices over the liver, will be most important remedies. The combination of calomel, antimony and opium, must be occasionally administered; and antimonials must be combined with the purgatives, which, in the form of pills, should be given to act regularly on the bowels, and should be aided occasionally by the sulphate of magnesia and other saline purgatives; and, in many cases, the saline purgatives, alternated occasionally with mercurials, are sufficient to cure the disease. A free and uninterrupted action from the skin is most desirable; and to promote this most effectually, the warm bath may be very advantageously employed; and the poultice, while it restrains the patient in bed, assists forcibly as a diaphoretic measure.

Under treatment of this kind, a very large proportion of cases are completely cured; and where the result is otherwise, it generally arises from some complication of diseases; most frequently from previous disorganization of the liver, or from neglect, on the part of the patient, in not applying for medical aid, or in not steadily pursuing the plan laid down; for it is not uncommon to find persons inclined to make light of an attack of jaundice, if the pain or inconvenience attending it is not such as to prevent them altogether from pursuing their usual occupations. Nothing, however, can be more injudicious; and it is the duty of the practitioner to impress this upon his patient; for it can never be a matter of slight importance that either obstruction or inflammatory action should exist in the intimate structure of a secreting organ, and still less in an organ so delicately complex as the liver; and there is no reason to doubt, that although, even without treatment, an attack of this kind may pass off, the liver will be some time before it is completely recovered; and thus a tendency to relapse, or to a renewal of the complaint, may occasionally be observed, shewing itself at longer or shorter intervals throughout life, and terminating at length in the destruction of all the powers of the stomach, the ulceration of the colon, miserable emaciation, or universal dropsy.—*Guy's Hospital Reports*, No. III.—*American Journal of the Medical Sciences.*

---

17. *Pins taken from the Body.*—Two interesting cases of this are related in a recent number of the London Medical and Surgical Journal, (March 18, 1837.) The first case was that of a woman who had a packet of pins in her left bosom, and was quietly walking along, when a drunken man rushed against her with such force, as to drive the contents of the package into her left bosom; a good deal of hemorrhage immediately set in; a professional gentleman removed a few of the pins, but was obliged to leave the greater quantity in the organ they had entered so deeply. A few months after, she was admitted into the hospital. A cicatrix existed upon the surface of the left mamma, at the entrance, also the cicatrices of three or four incisions upon the upper part of the arm, and as many more upon the left side of the trunk, and upon the upper and posterior part of the left lower extremity. During her stay in the hospital, whenever a pin was about coming to the surface, a slight degree of redness of the integuments always preceded, and pain was felt in the spot, particularly upon handling it; with the greatest certainly of finding a pin, an incision would then be made, and the pin found four or five lines from the surface, which was readily removed with a forceps. Before she left the hospital, as many as twenty pins had been altogether removed, and that which was last removed, invariably made its appearance at a greater distance from the seat of the injury than the one which preceded it. Thus the last pins removed were from the dorsum of the left thumb, and the anterior part of the left ankle. In their course they invariably followed the intermuscular cellular intervals, and confined their route to the left side of the body, never passing the *median cellular boundary.*

In the other case, the individual was pinning up some bed curtains previous to finishing them, and this operation requiring more hands than one pair, some of the servants volunteered their services, and amongst the rest a smart dapper footman. This gentleman's attention being more directed to the shape and figure of the principal, who was standing on a high stool, with her mouth full of pins, than to any use he was present for, gently commenced paying his devoirs, by giving a portion of her gluteous maximus—which was in the most inviting position for such an operation—a smart embrace between the finger and thumb of his right hand. She started, her foot slipped, her ankle was strained, but, what was of more serious consequence, she swallowed the pins! The poor girl suffered great pain and fright; a physician was immediately sent for, who removed as many pins as he could from the bag of the pharynx. She was admitted into hospital. Several pins in addition were brought away; and she left, in about two months, in the full conviction that at least two dozen pins were distributed in various parts of her body. For a year afterwards she was a constant visiter at hospital, to have pins removed from various parts of her body. Unlike the first case, the pins followed no regular boundary; which is to be accounted for by their entering the bag of the pharynx in every direction.

*Ibid.*

---

18. *Treatment of Hydrocele by Injections of Iodine.*—M. Velpeau prefers a solution of iodine to wine as an injection for the cure of hydrocele. He employs the tincture, in the proportion of one to two drachms to an ounce of water. Having emptied the cyst by puncture, he injects from one to four ounces of this liquid. It is not necessary to fill the tunica vaginalis, provided the tumour is pressed so that the medicament is applied to the whole of its interior. The fluid is then withdrawn, but without fearing to leave a small quantity. As it is not necessary to fill the cyst or warm the fluid, a common urethra syringe answers for the injection. If the hydrocele is large, it may be necessary to repeat the operation three or four times. After the injection the part swells for three or four days, but without causing fever or severe pain; resolution then takes place rapidly. M. V. has successfully treated twenty cases by this method.—*Archives Generales*, January, 1837.—*Ibid.*

19. *On Pessaries, and the Radical Cure of Prolapsus Vaginæ et Uteri.* By Prof. Dieffenbach.—This distinguished surgeon has long discontinued the use of pessaries in his own practice. To them he ascribes the occurrence of many diseases of the vagini and uterus, as well as of the neighboring parts; and although he admits that there may be cases in which their use is likely to be beneficial, he considers that such cases are comparatively very rare. He was led to adopt the mode of practice which he here recommends, by seeing the case of a woman, the subject of prolapsus of the vagini and uterus, in whom parts of the vagina sloughed, during its state of prolapse: the uterus and vagini were replaced whilst granulation was going on, and the result was a complete cure of the disease. The first case with which Dieffenbach met, after this, on which he was determined to imitate the natural process, was that of a woman with prolapsus of the uterus, which could be easily replaced, but as easily prolapsed, when it was not kept in by a sponge.

The operation was thus performed. The bladder and rectum were emptied; the uterus was made to prolapse, and a portion of about the size and shape of a hen's egg was removed from the left side of the vagini, the sharper end of which was directed backwards, the opposite end forwards, and came in contact with the nymphæ. The fold was then seized with a pair of forceps, the uterus being previously pressed somewhat backwards to take off the tension of the vagini, and then dissected out with a slightly curved scalpel. The same process was repeated on the right side. The wound was cleansed, and at its hinder part two sutures were applied, the uterus was next replaced, and three other sutures were applied within the vagini. Had all the sutures been completed before the attempt was made to replace the uterus, it is possible that its reduction could not have been effected. Some little irritation followed, which ceased, however, on the removal of two of the sutures from either side. On the sixth day, all the sutures had separated.

Since the time at which Dieffenbach performed this operation, he has repeated it very often. He now employs a smaller number of sutures; usually only two, and never more than three. In many cases he uses no sutures at all, as the borders of the wound in the vagini mostly lie low in contact after the uterus has been replaced. The suture is required where there is great relaxation, and a want of irritability of the vaginal membrane; on the other hand, when the individual is robust and the vagini thick, it is better to dispense with sutures. When the surface of the vagini is mortified, it is necessary to fill it with charpie. Tepid mucilaginous injections should be used for some days, and after these, cold water. If, when cicatrization is going on, there is no evident narrowing of the vagini, a compress of charpie smeared with a resinous ointment, and the repeated application of the lapis infernalis, should be employed.

Dieffenbach has often removed the fold from the vagina after having replaced the uterus, by drawing a portion of the former outwards, and cutting it off by a knife, with a sawing motion. This is a far easier mode of operating, but great care is necessary not to injure the bladder or rectum, which may happen if the fold of vagina, when tightly stretched by the forceps, should be cut off too near its base. Sutures are not employed in this case.

The position of the patient in the operation above described, should be the same as that for lithotomy. The state and relations of the rectum and bladder with the vagini and uterus should be ascertained previous to the operation; of the former, by means of the finger; of the latter, by Desault's silver catheter. The catheter sometimes draws off a quantity of retained urine; the evacuation of the bladder being often rendered very difficult by the prolapse of the uterus.—*Medicinische Zeitung*, No. 31, 1836.—*Ibid.*

---

20. *Treatment of some forms of acute Opthalmia by the application of successive Blisters upon the Cutaneous Surface of the Eyelids.*—The sudden disappearance, on the occurrence of erysipelas of the face, of some forms of opthalmia, which has long resisted the usual means of treatment, first led M. Velpeau to the use of blisters in ophthalmia, applied as near as possible to the inflamed part, and therefore upon the eyelids. The advantages resulting from their use, were found to be very considerable, and to be most evident in those cases where the inflamed vessels were not the same as those the action of which was increased by the use of the blisters; thus, e. g. inflammation of the cornea, the vessels of which are derived from the ciliary branches of the ophthalmic artery, is more benefitted by blisters than inflammation of the internal surface of the eyelids, which are supplied by the palbebral branches, and which are directly acted upon by the new cause of irritation. M. Velpeau therefore thinks that blisters applied upon the eyelids will be of the most service in those cases where the inflammation is nourished by the muscular branches of the ophthalmic artery, the ciliary and central of the retina. M. Velpeau has now employed blisters in these cases more than fifty times. In no case have they increased the evil which they were intended to remedy, they have not increased the pain, they leave no indelible marks upon the face, and the only evil effect which has been observed to follow them, is an occasional stye.

The immediate advantages following blisters in such cases, are—diminution of headache, if it previously existed; diminution of lachrymation and intolerance of light, of redness and thickness of the ocular conjunctiva; cleansing of ulcers; lessening of the cloudiness and suffusion of the cornea and aqueous tumour, of effusions of pus or lymph, or at least a discontinuance of their formation, together with improvement in the general state of the patient.

But in many cases, there is a class of secondary effects, which do not clearly manifest themselves before the blistered surface begins to heal. Of these, the most remarkable is the diminution of the cloudiness of the transparent parts of the eye. If lymph be deposited at the bottom of an ulcer; in the substance of the cornea; under the form of hypopium; in layers of masses, it is equally under the power of the blister, disappears as it were by enchantment; so that the clarification of the cornea and aqueous tumour appears to be the special object of the blister. Another effect, almost as constant but not so rapid as the preceding, is the extinction of inflammation the conjunctiva, then in the cornea. If there is chemosis, this gradually diminishes. Should ulcers be formed on the cornea, when the inflammation is calmed, there will require other remedies to hasten their cicatrization. Blisters applied in front of the orbit are not beneficial in all forms of ophthalmia. They are of especial advantage in favoring the absorption of matters which tend to obscure the clearness of the transparent media of the eye; and their use is indicated in acute inflammations, foreign to the eyelids; in inflammations of the various tunics of the eye, and of the parts contained within the orbits. In opthalmias which are seated in the fibro-serous tissue of the eye, i. e. in rheumatic ophthalmias, the effects of blisters are more complete than in any other cases, whatever may be the intensity of the inflammation. No topical application is so efficacious. The disease is, as it were, extinguished beneath the blister, and ordinarily disappears entirely by the use of one or two blisters in the space of from eight to fifteen days. The catarrho-rheumatic ophthalmia is still more under the influence of the blisters, applied upon the eyelids. M. Velpeau concludes his paper by hinting at the possible advantage to be derived from blisters in the earliest period of cataract; he grounds the idea of their possible utility, on the influence which they appear to possess of restoring those parts of the eye which have become cloudy, to their natural transparency; but the notion is supported by no facts.—*British and Foreign Med. Rev.*, from *Jour. des Connaissances Medico-Chirurgicales.* September, 1836.—*Ibid.*

---

21. *Prof. Rust's method of operating for Cataract by reclination.*—When Prof. Rust was a student at the University of Krakau, he became acquainted with an empiric who was celebrated for his success in operating for cataract, although totally unacquainted with the anatomy of the eye, or the general principles of surgery. This man operated with a *round* needle, mounted on a clumsy metalic handle; yet seldom his cases presented those secondary accidents of inflammation, &c. under which the operations of more skilful surgeons often fail. His acquaintance with the young student gave him an opportunity of acquiring the anatomical knowledge in which he was deficient, and after a retirement of some months he sallied forth on another campaign, proud in his increased knowledge, and armed with fine, double-edged needles of the most approved construction. But the quack's good fortune had forsaken him; his operations were now followed by a multitude of accidents which he had never before witnessed, and several of his patients lost their eyes from inflammation and suppuration. Under these untoward circumstances, he prudently determined to abandon the path of science, lay aside his double-edged instruments, and resume his old, clumsy, blunt needle. His operations were then as successful as they had been in the first instance. The reflective mind of young Rust was excited by this remarkable coincidence, and he soon discovered that the whole secret depended on the man's employing a *round* needle, which never produces the same degree of irritation as the cutting needles employed by most surgeons. Further experience confirmed the truth of this fact, and led Dr. Rust to propose the following method of operating, which he has since practised with so much success himself.

*Method.*—The instrument used by Professor Rust is a fine, round needle, flattened a little at the point only, for the purpose of penetrating the tunics of the eye, but not broader here, or double-edged; half the blade, next the handle, is gilt, or otherwise stained, that the operator may be able to judge more accurately how far the needle penetrates into the eye. The patient's head being properly placed, and fixed by an assistant, the operator depresses the under eyelid, and having placed his needle a little in front of, and parallel to, the cornea, with the point corresponding with the centre of the cataract, he thus estimates how much of the blade must penetrate the eye before the point can reach the centre of the lens. Having well calculated this, he passes the point of his needle into the conjunctiva (in a direction which would traverse the optic nerve if continued,) about one line behind the edge of the cornea, and from one half to a whole line below the transverse diameter of the eye, and sinks it into the eye until it has reached the depth calculated on after the first manœuvre. The handle of the instrument is now directed backwards, which brings the point forwards, and makes it penetrate to the depth of half a line; the posterior surface of the capsule, and the middle of the lens, is at the back part.—Having thus penetrated the lens, it remains to recline it, which Professor Rust executes by simply turning the handle of the needle, and bringing it a little back; in this way the anterior surface of the lens is directed outwards and downwards; its posterior surface inwards and upwards; its upper edge forwards, and its under one backwards. The operator now endeavors to push the depressed and reclined lens backwards and outwards, by bringing the handle of the needle forwards and a little inwards towards the nose; this done, he withdraws the instrument in the direction it has now assumed. Unless the latter precaution be taken, we run the risk of bringing the lens again forwards; to prevent which, when the operation is finished, Prof. Rust sometimes dashes cold water suddenly against the patient's face, while he himself holds the needle fixed in the direction judged most convenient; the patient suddenly draws back his head, and in this manner withdraws the lens from the point of the needle.

The operation now described has the advantage of being extremely simple, and is seldom followed by any secondary accidents; the ciliary processes are uninjured, and as the capsule is opened, the chance of the lens being absorbed is much increased.

The operation of Doctor Rust is liable to two objections. First, that the operator cannot see what he does when once the point of the instrument is introduced. However, this is of less consequence, since the depth to which the needle penetrates can be estimated with so much accuracy. Secondly, that simple reclination of the diseased lens is never sufficient to produce absorption of that body, which must be moved to and fro in the vitreous humor. In the uniform success which attends Professor Rust's method of operating, is to be found the best answer to this latter objection.—*Lancet*, for March 11, 1837.—*Ibid.*

22. *On the Pneumonia of Old People.* M. M. Hourmann and Dechambre.—The following remarks on this important topic have been derived from a very extended series of observations, made chiefly in the practice of the great Hospice of the Salpetriere, in which there are usually two or three thousand poor old women. M. M. Hourmann and Dechambre have paid particular attention to the etiology of this affection, and the results of their enquiries are alike interesting and instructive.

The chief predisposing cause to the pneumonia in question is unqestionably to be found in the almost habitual bronchorrhea to which old people are subject. The mucous membrane of the bronchi is in a relaxed and weakened state; the secretion from its surface becomes more copious as we advance in life, and this indicates a tendency to congestion of its capilliary vessels; the membrane itself is less sensitive, and the submucous muscular or fibrous tissue is less irritable than it was wont to be; the cartilaginous rings of the air tubes are more unyielding—all these circumstances operating together must predispose the lungs to be readily affected by atmospheric changes, and will account for the extreme obstinacy and frequent danger of such affections in old age. But there are other conditions of the system at this period of life, which act in a similar manner. Such, for example, is the frequency of heart diseases, and the narrowing of the thoracic cavity in consequence of the enlargement of some of the abdominal viscera.

Considering, therefore; the influence of these predisposing causes, it is not to be wondered at that pneumonic disease is of great frequency, and of most tedious obstinacy in old age; and the circumstance of both lungs being so much oftener affected simultaneously in aged people than in those of earlier life can only be explained on the plea, that both lobes have undergone certain unfavorable changes, by which they are more predisposed than formerly, to be affected by morbid influences.

Of the occasional or existing causes of pneumonia in old people, by far the most frequent is the variableness of the weather. The influence of atmospheric conditions is well illustrated by the following table:

Of 156 cases observed in the Salpetriere during the course of one year,

| | | | | |
|---|---|---|---|---|
| 17 | occurred in | January | 6 | deaths |
| 19 | " | February | 14 | " |
| 31 | " | March | 20 | " |
| 20 | " | April | 9 | " |
| 11 | " | May | 3 | " |
| 6 | " | June | 4 | " |
| 3 | " | July | 2 | " |
| *1 | " | August | 1 | " |
| 1 | " | September | 0 | " |
| 5 | " | October | 4 | " |
| 19 | " | November | 15 | " |
| 23 | " | December | 10 | " |

*During the months of August and September, the cases were not so regularly registered as during the other months. There may have been therefore admitted two or three cases more than we have details of.

" As a remark of very general truth, it may be stated, that the variableness of the weather is much more apt to induce pneumonia and bronchitis, than mere severity of the cold..

From the preceding table, it appears that the months of March and April are on the whole most unfavorable. The pernicious influence of sharp cutting winds has been noticed by all statistical writers. Hippocrates alludes to the frequency of chest disorders during the blowing of North-East winds. M. M. Hourmann and Dechambre, confirm the truth of this observation.

"Ce que nous pouvons assurer relativement á nos vieillards, c'est que dans le cours deux premieres années et cette année meme (1836), au mois de Mars et Avril, pedant tout le temps (douze jours en 1834), qu'une augmentation considerable eut lieu dans le nombre des pneumonies, la fleche placée sur l'eglise de la Salpetriere a pris la direction du Nord-Est et s'y est maintenue invariablement. Nous avons dit qu'à cette epoque aussi la temperature etait abaissée et soumise à des variations."

The influence of long decubitus in bed ought certainly to be enumerated among, if not the inducing, at least the predisposing causes of pneumonia in old people. Hitherto the influence of this circumstance has not been sufficiently attended to by medical enquirers. That the statis of the blood, in those parts of organs which are kept for a length of time in one position, has a tendency to induce, or at least to favor the accession of a troublesome sort of inflammatory action, is strikingly illustrated in the case of old people who have been long bed-ridden.

From the researches of M. M. Hourmann and Dechambre, it appears that the posterior and inferior parts of the pulmonary lobes are much more commonly affected with *intervesicular* pneumonia than the anterior and upper parts; that *vesicular* pneumonia is more frequent in the anterior than in the posterior edges of the pulmonary lobes; and that pneumonia of the superior lobes has in most cases its seat all around the fissure, which separates them from the middle lobes, and pneumonia of the lower lobes is very generally seated in their posterior parts. This predilection of pneumonia for those parts which are most "declives" can be fairly attributed only to the mechanical accumulation of the blood.

Our authors state also that pneumonia of the lower lobes is much more frequent on the right than on the left side, and account for this by saying that most bed-ridden people usually lie on the former side.

The influence of prolonged decubitus in determining the seat of pneumonia in old people has been much insisted upon and very ingeniously discussed by M. Piorry in his memoir on what he calls "pneumonie hypostatique."*

---

*There is a notice of M. Piorry's memoir in the number of this Review for July, 1833. As the subject is but little known, we shall make one or two extracts from it. "It is well known to anatomists, that the posterior parts of the lungs are almost always found on dissection to have a deep purple venous color, while the fore parts of the lobes are pale and speckled. This appearance of congestion is correctly attributed to the subsidence of the blood after death towards the most depending parts, and constitutes the "engouement cadaverique" of the French pathologists. Now M. Piorry is of opinion that in enfeebled states of the system, especially in

To sum up in a few words the doctrines of M. M. Hourmann and Dechambre on the etiology of senile pneumonia, it may be stated, that some of the causes, as for example weakness of the system, and tendency to chronic catarrh, operate on all the patients; that other causes, as diseases of the heart, are present in a large number; and lastly, that those patients who are bed-ridden, are on the whole more susceptible of being affected by atmospheric vicissitudes, than those who are able to be moving about.

The accession of senile pueumonia may take place in two very di-s tinct forms. It is either sudden, setting in with a chill, pain of the side, as is ordinarily the case in young subjects; or it may creep on insidiously, and establish itself, before its existence has been suspected, and without the presence of any well marked symptoms. At the Salpetriere, senile pneumonia assumes almost always the first of these fevers during the months of March and April. The shivering and the attack of pain are often preceded for several days by headache, general langour, coryza with or without epistaxis, flying pains in the limbs, &c. and the pneumonia being once fairly established, the patients soon fall into a state of stupor and adynamia.

In the second mode of attack, there is neither shivering, nor any pain in the side at first; the patient only feels ill at ease, and altogether more feeble than before; the breathing is hurried and sometimes irregular; there is a paroxysmal cough, accompanted with difficulty in expectorating the phlegm, the skin is warm, and the tongue is dry. Such are often the premonitory symptoms of pneumonia in old people. But even these, now enumerated, are not always present. There may be neither cough, nor any disturbance of the breathing, even although the patient had been, before the attack, subject to an asthma, or chronic catarrh, and the only symptoms may be a general malais and feebleness. This most unexpected occurrence has been repeatedly noticed by M. M. Hourmann and Dechambre, and from its very singularity, it becomes a

---

aged people, a tendency to the same subsidence may take place during life, when the patients are long confined to the horizontal posture, and may thus, if it does not actually prove an exciting cause, at least much aggravate any inflammatory affection of the lungs. Eight illustrative cases are adduced; they occurred in old people admitted into the Salpetriere as objects of charity.

Professor Boyer long ago remarked that it was most injudicious to confine old people for any great length of time to bed: and even, when it is absolutely necessary, as after fractures, or other severe injuries, they should be told to sit up occasionally in bed, in order to prevent the congestion of blood, not only in the skin of the back, but also in the depending parts of the internal viscera." p, 212.

The following notice of the sentiments of another estimable French physician may be appropriately introduced here.

M. Bricheteau has of late met with several cases of fatal pneumonia, characterised on dissection by a ramollissement of the pulmonary tissue, which he calls "splenification," from its resemblance to softened spleen. In all the cases, there had been a considerable impediment to the free circulation of blood throughout the lungs, in consequence generally of an associated heart disease. The tissue had become, as it were, soaked with blood, and so much softened, that it was easily lacerable, and reducible into a soft pap on pressure.

This sort of pneumonia is much more frequently met with in the old and enfeebled, than in younger patients; and is generally associated with disease of the left side of the heart.

valuable symptom to the experienced practitioner. In reference to the dyspnœa, our authors very properly inculcate on their readers the propriety of not trusting to the mere accounts of the patient. The physician ought to examine the breathing for himself, to count the number of respirations, to percuss the chest, and to ascertain the sounds elicited on auscultation. With all the assistance to be derived even from these means of exploration, it is occasionally not easy to form an accurate diagnosis of the case, and to prognosticate its probable result. Take, for example, such a case as the following:

One of the old inmates of the Salpetriere rises at her usual hour, makes her own bed, eats her food as usual, and moves about the ward; in the course of the day she feels fatigued and exhausted, lays down on her bed, and, after a short struggle of difficulty in breathing, expires. "C'est la une des *morts* dites *subites* de vieillesse à la Salpetriere." On dissection, the physician is surprised to find a large portion of the pulmonary parenchyma in a state of suppuration. Some remarkable cases of this description have been observed by M. M. Hourmann and Dechambre, in old women admitted into the Infirmary of the Salpetriere for other diseases. For example, a poor Vieille, 85 years old, had been admitted a few days before for a stomach complaint: she was quite gay and cheerful, and to show her good appetite to the doctors, she ate a piece of hard biscuit in their presence.

The visit to the infirmary was not over, when one of the nurses came to inform them that their old patient was dying. They arrived just in time to see her expire; some of the buscuit crumbs still hung around her mouth. On dissection, the entire substance of the lower lobe of the right lung was found in a state of free suppuration.

M. Falret mentioned a similar case to the authors. An old insane woman, remarkable for her habitual loquacity and strength of voice, very suddenly fell down, and died in the course of an hour or so. The whole extent of the right lung was found in a state of grey hepatisation.

A very important conclusion, which M. M. Hourmann and Dechambre have deduced from their researches, is, that senile pneumonia very generally commences and advances in an obscure latent form, when there is co-existent cardiac or cephalic disease—very common complications—and that on the other hand the attack is usually sudden and acute, in persons who are free from such diseases.

The *symptomatology* of senile pneumonia may be, in part at least, inferred from the preceding remark. The pain is seldom sharp or severe; and this is often the case, even when the corresponding portion of the pleura is inflamed at the same time. The patient complains of a mere sense of soreness or uneasiness over the entire affected side. In those cases in which the disease commenced with a stitch, this seldom lasts above two or three days, and then it is replaced by a diffused obtuse pain. The dyspnœa is often as unsatisfactory a symptom as the local pain. The breathing may be not at all embarrassed; usually it is accelerated; but in some cases it is even slower than usual. It has been observed by our authors, that the dyspnœa is always much less severe when the disease affects chiefly the lower lobes. When the upper lobe or lobes are inflamed, the breathing is almost invariably much distressed, and often constant orthopnœa is present. Occasionally, however, the inflammation of the upper lobes commences in a latent form, and all the symptoms remain, throughout, obscure and indistinct.

What we have said of the pain and dyspnœa, may be extended to the cough, as one of the symptoms of senile pneumonia. It is sometimes so inconsiderable, that the patient scarcely is aware of its presence.

He has perhaps only two or three fits in the course of the day; and these are apt to come on only when he makes any exertion, or changes the temperature in which he is in. The expectoration, also, in not a few examples, affords no evidence of the existing disease.

We have already remarked, that M. M. Hourmann and Dechambre have repeatedly observed, that in those cases where the patient had been subject to a chronic catarrh before the accession of the pneumonia, the expectoration often ceases altogether.

With respect to the auscultatory symptoms of senile pneumonia, they are not, on the whole, so distinct and satisfactory as in the pneumonia of younger people. The genuine râle crepitant is rarely to be heard; mucous and sonorous râles are the predominating sounds. Sometimes the râle has an unusually dry resonance, and resembles the crackling of parchment rubbed between the fingers. The respiratory bruit is almost always more or less bronchial in the neighborhood of the affected parts. The complete extinction of the respiration over the parts themselves is a much more rare occurrence in the old, than in the young and middle aged, affected with pneumonia.

It particularly deserves to be noticed that, in some cases of senile pneumonia, ausculation affords no evidence of its existence. The words of our author are—

"Nous avons vu des pneumonies, chez nos vieillarde, traverser toutes leurs periodes, sans donner lieu a aucun râle. Alors ou bien l'on n'entendait rien, ou bien le bruit respiratoire etait tres fort et pure. Nous avons recueilli un fait de ce genre, meme dans un cas de pneumonie de tout le lobe superieur droit, et l'auscultation a été pratiquée jusqu'à l'instant de la mort."

During the progress of senile pneumonia, the functions of the cerebrum are very generally disturbed; the patient becomes delirious, and the delirium lapses more or less speedily into coma. The period, at which the mental disturbances usually commences, is when the process of suppuration is about to be established: sometimes, however, the invasion is much earlier. In the greater number of cases, there is no delirium nor coma; but only decay and hepetude of all the intellect and moral powers. Without being positively irrational, the patients appear to be quite lost; they do not understand what is said to them; their answers have, perhaps, no reference to the questions put, and they remain taciturn, and take no notice of any thing. In short, they lapse into that condition so emphatically described by Shakspeare as "second childishness and mere oblivion."

The symptoms afforded by the state of the tongue, of the skin, the bowels, and the urine, are far too uncertain and unsteady to warrant any confidence being placed in them. The pulse itself is apt to misguide us, if we attend too much to its state. That the very essence, if we may so speak, or the proximate cause of the disease in old persons, is different from that of pneumonia in younger subjects, is rendered probable by the circumstance of the blood, when drawn in the former set of cases, being generally without any buffy coat. Of 24 venæsections, practised by M. M. Hourmann and Dechambre, the blood was buffy eight times only; and in 23 other cases, the blood was buffy in nine only of them. Of 17 cases, in which the blood was found to be buffy, the invasion of the disease had been acute in 15, and latent in two. Of the 30 cases in which it was not buffy, the invasion had been latent in 11, and acute in 18 of them.

The physical characters of the coagulum are, in general, very different from those exhibited by buffy blood. It is not red and firm, but soft, black, or even of a greenish hue. Sometimes the blood does not form a proper clot, but assumes the consistence of molasses.

M. M. Hourmann and Dechambre very properly remind their readers of these facts, when they come to treat of the appropriate remedies for senile pneumonia. Free bloodletting must be practised with great circumspection; the powers of life are easily made prostrate, and if once permitted to be so, there is little hope of reanimating them. They tell us that they have sometimes found it necessary to exhibit wine and other stimulants, at the very time they were drawing blood. Perhaps, under such circumstances, it would be more judicious to employ local depletions, by cupping, than general evacuations.

Emetics are highly praised by our authors. They have no faith in the contra-stimulant practice of exhibiting large doses of tartar emetic. Blisters, large and applied directly to the chest, are among the most efficacious remedies; in most cases, they may be used with advantage from the very commencement of the disease. [No allusion is made to the use of expectorants: the judicious use of oxymel of squills, compound tincture of camphor and syrup of poppies, in the form of a mixture, will be found of sovereign benefit in most cases.—Rev.]—*Archives Generales de Medicine.—Medico-Chirurgical Review.*

---

23. *Morbid Anatomy of Typhus Fever in Paris, Geneva, Dublin, Glasgow, Liverpool, Manchester, and London. By* Dr. Lombard, *of Geneva.**—The following facts observed by Dr. Lombard, are calculated to give rise to useful reflection in the mind of the pathologist and of the practical physician.

In Paris and Geneva Dr. Lombard has either made or seen a great number of dissections of patients dead of typhus fever. In all of them, without exception, he has found enlargement of the aggregated follicular glands in the lower third of the ileum, attended with ulcerations of the mucous membrane. So constant is this post mortem appearance on the Continent, that it is looked on as an essential element in fever.

With this experience, Dr. Lombard went to Glasgow. The fever cases there were similar, almost identical, in character, to those he had witnessed abroad. A patient died, and he entertained no doubt of discovering follicular disease. There was none.

---

*Dublin Journal, Sept., 1836.

When Dr. Lombard arrived in Dublin, he again had opportunities of examining the bodies of fever patients. He inspected two, but found no follicular disease. He was informed by the physicians of Glasgow, that such lesions occur in about one-third of the bodies examined after death. The Dublin physicians told him that the alterations are even less frequent in that city, particularly during the present epidemic, which has preserved nearly the same characters for the last eighteen months. In former epidemics those changes appear to have been more common.

In Liverpool, where there were many cases of fever, Dr. Lombard found them similar in character to those of Dublin. The morbid appearances were similar too. Serous effusion in the brain, meningitis, pneumonia, and occasionally some injection of the mucous coats of the intestines, and occasionally also ulcerations of the ileum and cæcum, varying in frequency with the different seasons, composed the catalogue.

In Manchester, the total number of typhous patients received into the fever hospital in the year did not exceed three hundred and thirty-two, a proportion far inferior to that observed in Liverpool and Glasgow. Dr. Lombard was informed that it was by no means constant for intestinal ulcerations to be found in the bodies of the dead.

In Birmingham, the fever cases are not numerous, but Dr. Lombard learnt that ulcerations of the lower part of the ileum were invariably found.

In London, the annual number of admissions into the fever hospital has been diminishing for the last three years. In 1835 the number of typhus cases was 250. Serous effusion in the ventricles and other morbid appearances of the brain, are more frequently to be met with, than the abdominal complications which in the shape of ulceration are not to be found in more than one fourth of the cases, according to Dr. Tweedie's researches; and this proportion of ulcerations in the lower part of the ileum varies with different seasons, being much greater in autumn than at any other period of the year. The continued fever of London is very different now from what it was some years since; it was then highly inflammatory, and local and general blood-letting could not then be neglected; while now the symptoms of depression and weakness have taken the lead, and so low is the present fever, that wine, camphor, and spirit of mindererus are to be given in almost every case.

Thus we have seen that in Birmingham only are intestinal ulceraations said to be constant. Dr. Lombard proposes an ingenious hypothesis, to account for the discrepancy in the morbid anatomy of fever here and on the Continent. But it is too unsatisfactory to induce us to go into it. On one point, however, we find it difficult to agree with Dr. Lombard; and that point is no trivial one. We are not sure that follicular enlargements or intestinal ulcerations *do* attend all typhus fevers abroad. In support of this observation we may refer to M. Andral himself, who classifies "ataxo-dynamic," or typhus fevers thus:—1. Those in which the follicles are altered. 2. Those in which there is simple inflammation of the gastro-intestinal mucous membrane; and, 3. Those in which *no* lesion of the digestive tube exists.

M. Louis, indeed, has chosen to restrict the term typhus to cases of the first class. But it may be questioned whether this is not, even in France, an arbitrary nosological definition, rather than an accurate expression of the fact.

---

24. *Contagious Typhus.*—Dr. Perry, one of the physicians to the Glasgow Infirmary, and a man of observation and candor, has published some remarks on this subject in the Dublin Journal. His paper is in the shape of a criticism on that of Dr. Lombard, of Geneva, which will be found in our present number. We need not enter on the points discussed, but confine ourselves to stating the results of the personal observation of Dr. Perry.

He had, for some years, entertained the opinion, founded upon an extensive series of observations, that contagious typhus is an *exanthematous disease*, and is subject to all the laws of the other exanthemata; that, as a general rule, it is only taken once in a life-time, and that a second attack of typhus does not occur more frequently than a second attack of small pox, and judging from his own experience, less frequently than a second attack of measles or scarlet fever. He ridicules the idea that contagious typhus may originate spontaneously from filth, bad diet, crowding, &c., and terms such notions "fancies, contrary to sound philosophy." There is certainly some plausible evidence extant, in support of opinions of the nature that he scouts, but Dr. P. openly professes his utter want of faith. Yet he is not so sceptical as might be expected upon other points. He is satisfied, for example, that typhus fever is not contagious before the ninth day, or perhaps not till a later period than that. He thus states the reasons for this supposition:—

Among many circumstances which establish this opinion, I may mention one experiment which I made upon a pretty extensive scale.—The fever wards of the Glasgow Royal Infirmary are each capable of containing twenty patients. The beds are arranged in two opposite rows, and are pretty near each other. While the patients are in the acute wards, they are not allowed the use of their clothes, though they may be able to set up; they are, therefore, almost constantly confined to bed, excepting when rising to stool; and there is about one close stool to every three patients. Into the fever house are admitted cases of measles, scarlet fever and small pox; and patients are very frequently sent in, laboring under bronchitis, pneumonia, erysipelas, and other local inflammatory affections. I found, by experience, that when the latter class of patients were sent to the convalescent ward, where they necessarily mixed with the others, almost all those who had not a previous attack of typhus fever were either seized with it before leaving the house, or returned soon after their dismissal laboring under it, the period intervening between the time of their being sent to the convalescent ward, and the attack, never being less than eight days. Although means were taken to keep those recovering from small pox, scarlatina, &c. in a separate room from those convalescent from typhus, the rooms being adjoining, the non-intercourse was incomplete, and the result was, that these diseases occasionally spread among the typhus convalescents, and the convalescents from small pox and scarlatina caught typhus. In consequence of these observations, I adopted the practice of not sending, as formerly, to the convalescent wards, those patients affected with inflammatory diseases, unless I ascertained that they were secured against the disease by having had a previous attack of typhus; but kept them in the acute fever wards till they were so far recovered as to go to their own houses, and the result was, (and the practice was continued for several months,) that not one of those detained in the acute wards caught the disease while there, or returned with it afterwards. From the above, and other observations, I have adopted the opinion, that typhus, like measles, small pox, &c. is chiefly spread during the period of convalescence."

Dr. Perry recommends the universal adoption of the practice of shaving the head of every fever patient admitted into the Glasgow Infirmary, and of washing well in a warm bath every such patient, when dismissed. We are gratified to learn that he is now preparing statistical tables to illustrate points connected with fever. Its prevalence in Glasgow, and his situation at the Infirmary, will render such tables at his hands very valuable.

---

22. *On the Motions and Sounds of the Heart.* By Drs. WILLIAMS, TODD and CLENDINNING.—The memoir on this subject was read at the Bristol meeting of the British Association, and is of considerable length. The conclusions arrived at are supported by many experiments on living animals, which are detailed. We gave a brief notice, in our fourth number, of the conclusions come to by the gentlemen above named, who constituted a committee in London appointed for the investigation of this interesting and important subject. We give here, in the words of Dr. Clendinning, the reporter of the committee, the summary and conclusions which terminate the report:

1. The first sound of the heart, as heard in the chest, is generally complex in its nature; consisting of one constant or essential sound, and one perceptible only under certain circumstances. This constant element of the first sound may be considered as intrinsic, appearing to depend on the sudden transition of the ventricles from a state of flaccidity in diastole to one of extreme tension in systole; [in a word, the first sound is essentially a muscular sound:] while the extrinsic, or subsiding sound, which in a variety of circumstances contributes largely to the first sound, arises from the impulse of the heart against the parietes chiefly of the thorax.

2. The collisions of the particles of the blood amongst each other, or against the interior parietes, valves, &c. of the heart, do not appear to have any share in the normal first sound of the heart; neither do the motions of the auriculo-ventricular valves; and the attrition of the opposite interior surfaces of the heart's cavities seems purely hypothetical.

3. The principal, and apparently only cause of the second normal sound of the heart, is the sudden closure of the sigmoid valves, by the columns of blood that recoil back on them during the diastole, impelled by the elastic contraction of the arteries.

4. The columnæ carnæ appear to act simultaneously with the parietes of the ventricles, and in such a manner as to make it apparently impossible that the auriculo-ventricular valves should close with a flap, in the same manner as the sigmoid valves.—*Med. Gaz., Dec.* 10, 1836.—*Brit. and For. Med. Rev.*

23. *On the Chemistry of the Digestive Organs.* By R. D. Thomson, M. D.—From experiments and observations made by himself and others, Dr. Thomson concludes that the stomach, in a state of health, retains a quantity of free muriatic acid, and also that dilute muriatic acid is capable of producing, by digestion with animal matter, at a temperature of ninety-eight degrees, a substance similar to chyme; and infers that this acid is therefore an important agent in the process of digestion. The only diseases of the stomach indicated by chemical reagents are common acidity, or heartburn, and pyrosis. In this last disease, Dr. Thomson has ascertained that the gastric secretion is diseased; alkali having taken the place of the free acid. He found the alkali to be ammonia, with (probably) also a little soda. In consequence of this, Dr. T. has prescribed acid, and he assures us with great benefit, immediate relief being afforded by it. In one case, of three months' duration, the use of an acid mixture for two days effected a perfect cure. In chronic cases, Dr. T. also prescribed anodynes, as conium and hyoscyamus.

In examining the fluids of the mouth during health, Dr. Thomson, with Donné, found them alkaline or neutral. Donné says that the mucous membrane of the alimentary canal (which is alkaline,) and the skin (which is acid,) constitute a kind of voltaic pile; when one of the poles of a delicate galvanometer is placed in contact with the mouth, and the other with the skin, distinct electric currents are produced, which cause the needle to deflect fifteen, twenty or thirty degrees. Dr. Thomson says, that he has found the mouth indicating an acid reaction whenever inflammation existed in any of the membranes in connection with it, as in laryngitis, pleuritis, bronchitis, gastritis, enteritis, &c.; and that inflammation of mucous and serous membranes generally is attended by the secretion of free acid. He therefore suggests the local application of alkaline solutions, where practicable, as in erysipelas, urethritis, &c.—*Records of General Science, Dec.* 1836.—*Ibid.*

24. *Physical Signs of the Height of the Diaphragm in the Chest.* By Edwin Harrison, M. D.—Dr. Harrison, who has been long engaged in investigating the physical signs of internal diseases, announces the following as among his recent discoveries. It is, however, but justice to Dr. H. to say, that he wishes his statements to be considered as mere generalities, and, as such, open to exceptions, limitations, &c.

1. A depression about the height of the lower dorsal or first lumbar vertebra, from which the finger, in mounting along the spine, will come to another depression, corresponding with tolerable accuracy to the height of the diaphragm at that part.

2. On moving the hand along either side of the chest vertically, on what may be considered as the median line, it will sink into a depression corresponding to the height of the diaphragm on that side.

3. A depression, or depressions, between the ribs can be felt or seen, or both felt and seen, at each abdominal inspiration, indicating (at least in the physiological state,) the presence of the diaphragm in that part of the chest. By this means the height of the diaphragm on the left side can be judged of, to a certain extent, even through the heart.

Query: What relation do these last-named depressions bear to the tilting up of the chest, which occurs not merely at its lower part, but as high up, at least, as the lateral depressions previously mentioned?—*Med. Gaz., Dec.* 10, 1836.—*Ibid.*

---

25. *On the Causes of the Resonance in Percussion.* By JOHN CLENDINNING, M. D.—This valuable paper contains the details of a case of pleurisy, with the appearances on dissection, and the notes of various experiments made on the dead body, with the view of ascertaining the cause of the sound on percussion and the effect of different conditions in modifying it. We can only here state briefly some of the results of Dr. C.'s enquiries, and must refer the reader to the interesting paper itself. The following are some of Dr. C's conclusions:

1. That in cases of hydrothorax, if either lung be nearly sound, there will be no fleshy dulness of resonance in any of the lateral parts of the chest.

2. That, in simple pleurisy, we must not expect to find the pulmonary resonance wholly wanting, even in cases of extreme effusion and compression.

3. That, in cases of effusion in the thorax, respiratory sounds may be expected to be heard at the usual places, so long as the effusion is not so great as to compress the lung to an extreme degree; probably so long as any considerable portion of the lung continues permeable by air of the bronchus.

4. That the wall of the chest is not the source of the soft pulmonary or dull hepatic resonance; but that this is owing to the lungs, liver, &c., respectively seated beneath the parietes; and that the walls are but conductors of the resonance of the organ beneath them.

This last conclusion is in direct opposition to the opinion of Doctor Charles Williams, as will be seen by the notice which immediately follows.—*Med. Gaz., Jan.* 7, 1837.—*Ibid.*

---

26. *On the Morbid Appearances in Death by Drowning.* By W. OGSTON, M. D. Aberdeen.—Dr. Ogston has endeavored to estimate, by the numerical method, the characteristic signs and morbid appearances of 17 cases of death by drowning, which came under his notice, during the last six years, in police practice. The bodies were examined in seven cases. The following are the principal results and remarks:

1. The only phenomena which were all but universal, were dilated pupils, clenched jaws, and semi-contracted fingers and thumbs. 2. There was commonly at first paleness of the face, which, after a longer or shorter interval, became livid. 3. Striking calmness and placidity of the features. 4. Excoriation at the ends of the fingers, and dirt or sand under the nails, which have been laid down as frequent marks of death by drowning, were not met with. 5. The tip of the tongue was usually found in accurate contact with the incisor teeth; in two instances included between the closed jaws, but not wounded. 6. In seven cases only was there froth about the mouth; an appearance on which much stress has been laid. 7. Froth was met with in the mouth in two cases only of those dissected; and in three only in the trachea. 8. Liquidity of the blood has been remarked; in five of the bodies examined, coagulated blood was found in the heart, though the great mass of blood was fluid. 9. Water was found in the stomach in almost all the cases examined.

M. Devergie has lately endeavored to show that, by certain appearances, the time may be determined which has elapsed since submersion; as might be expected, Dr. Ogston's cases do not confirm M. Devergie's statements.—*Edinburgh Med. and Surg. Journal, January,* 1837.—*Ibid.*

---

27. *On the Present State of Medicine in Italy.*—In the absence of a more formal Report, we are happy to lay before our readers the following extract of a letter, which we have just received from a very intelligent friend, a physician, who has been some time resident in Italy. We hope soon to present them with a more detailed account, both of the doctrines and practice of the Italians.

"From what I have seen of the Italian physicians, I would remark, that they display considerable learning and much acquaintance with ancient authors, yet in their practice they do not appear to advance beyond the days of Hippocrates or Galen. Following closely the footsteps of the great Father of Medicine, they carefully watch the natural progress of the complaint, but unfortunately they do little either to assist or counteract the operations of nature. In their practice they seldom employ any thing like decision or vigorous measures to cut short disease; and, even in the most acute complaints, they depend more on diet and regimen than on the use of medicines. I would not assert that they lose more patients than the English physicians; but I have no hesitation in saying that the bad effects of their mode of treatment is apparent in the immense number of chronic diseases which are continually presenting themselves, and which might probably have been prevented from becoming such, had depletion been more freely resorted to in the acute stage of the disease. Chronic gastritis, for example, with all the melancholy symptoms of dyspepsia which so frequently accompany this disease, and chronic enlargement and induration of the abdominal viscera, are every where to be seen.

"There is probably no point on which the continental practitioners differ from the English more than in the use of the lancet. Where an English physician would take away some pounds of blood to cut short an acute inflammation, an Italian takes away six or eight ounces, and repeats this venesection for days, until the disease terminates. Their ideas on this subject are very different from ours: we think that, the sooner we cut short an inflammation, the greater is our chance of preventing disorganization; they, on the contrary, believe that, by merely assisting the efforts of nature by small bleedings frequently repeated, they will at last subdue the disease more effectually, and without injuring the patient's constitution. Some, indeed, carry this difference of opinion so far as to assert that the great number of phthisical complaints in England is owing to the large bleedings which we prescribe, by which the powers of life are lowered to such a degree as to prevent the patient from again rallying. In their treatment of fevers, they employ purgatives much less frequently than we do: gentle diaphoretics, such as James's powder in one or two grain doses, alone or in combination with small quantities of spiritus mindereri and syrup of violets, are the only medicines they have any reliance on. The tepid bath is in great use among them in all febrile complaints, unless there be an eruption on the skin or an affection of the chest. The only purgatives which are used in fevers are magnesia and castor oil, the former most frequently; but, as I said before, they do not consider it so necessary as we do to clear out the primæ viæ. When they apply blisters in inflammatory complaints, (e. g. in pulmonic affections,) it is seldom over the part affected, but generally to the arms or legs.

"When they give medicines, they seldom, as in England, write out a prescription composed of a variety of ingredients, with directions for use: they generally write the name of one medicine on a piece of paper in the vernacular language, and give verbal directions as to the way in which it is to be employed. Indeed, their manner of prescribing is altogether so loose and irregular that one cannot help feeling that they do not place much reliance on what they prescribe.

"In regulating the patient's diet, however, they act with great judgment; here, indeed, they exhibit much more discrimination than the generality of English practitioners; and we may safely say that they are more indebted to their judicious management of the diet than to any medicine they give, for the cures they effect. In fevers and inflammations, not only every thing like solid food is withheld from the patient, but even the weakest broths are forbidden; lemonades, or asses' milk, diluted with two or three parts of water is all they allow, sometimes for twenty days, in fever; and it is only when the strength is giving way, or where the disease has nearly disappeared, that weak chicken broth would be permitted. In their theory of disease, they evidently lean more to the humoral pathology than to any of the more modern doctrines. This is remarkably the case in their treatment of chronic complaints, nervous affections, &c.; a great proportion of which they attribute to diseased humors in the blood, arising from repelled eruptions; and, for purifying the blood and removing these humors, they have a variety of medicines, chiefly vegetable, which they prescribe in the spring. Decoctions of fumaria, of graminia (the Fiorin grass), and of sarsaparilla, are often given for weeks or months, with this intention. The flesh of the viper is also a favorite remedy with them in eruptive diseases.

" In describing the opinions and practice of the Neapolitan and other Italian physicians, I must observe, that it is the older physicians whom I have chiefly in view; for there are many of the juniors in the profession who are fast approaching to the French or English mode of practice."

28. *Scientific Discoveries in Germany.—Extract of a Letter from* Prof. WEBER, *of Leipzig, dated Dec.* 1836.—At the Association of Naturalists at Jena, which I attended last September, a very interesting discovery (made by M. Fischer, of Carlsbad, and confirmed by M. Ehrenberg of Berlin,) was communicated,—viz. that *slate* (Polierschiefer), and likewise *flint* (Feuersteine), in great part consist of the shells or envelopes of petrified animalcules. Some species of animalcules have an envelope which contains silica, and therefore resists the action even of a glowing temperature. I have myself examined several varieties of slate; and, on comparing the animalcules I therein found with corresponding ones now in a living state, I satisfied myself that the same descriptions really occur very numerously in the above mineral. Ehrenberg has reversed the experiment, by evaporating (to dryness) mud in which these animalcules abound, and then burning it in a porcelain stove. He thus produced an artificial stone, which, on being examined, was found to contain the envelopes (Panzer) of the animalcules in the identical state in which they manifested themselves in the natural mineral.

Another remarkable discovery was that of M. Goppert, of Breslau; viz. that portion of plants, steeped in a metallic solution, imbibe the latter to repletion. If afterwards submitted to a glowing heat, the metal becomes reduced, and represents the precise form of the portion of plant, down to its minutest parts. The same experiment may be made with earthy solutions, and an artificial petrifaction thus be obtained.

You will remember being with us at the St. James's Hospital, when my brother and I proved, by experiment on the dead subject, that the head of the femur is retained within the acetabulum, or socket, *not* by the ligaments and muscles, but by the power of atmospheric pressure.* To these experiments we have since made an addition. Having sawed out the entire acetabulum, together with the upper part of the femur, (without injuring the ligaments,) from a dead body, we suspended the whole portion, by the acetabulum, from the lid of the bell appertaining to an air-pump, and attached a weight of two pounds to the femur. On exhausting the bell to within two or three inches, the head of the femur sinks to the extent of half an inch. On then readmitting the air, the head rises back again into the socket, despite the weight with which it is clogged. The same phenomenon manifests itself when circular division of the capsular ligament has been previously made; in which case, the upper half of that ligament performs the office of a valve. If the weight attached to the femur far exceed two pounds, the head of the bone delapses to the utmost extent that the ligamentum teres will admit of.—*Ibid.*

*See an account of the experiment, as made in Germany, in our third number, p. 236.—MED. REV.

